# Supplementary material for: Ultrafast transition from coherent to incoherent polariton nonlinearities in a hybrid 1L-WS2/plasmon structure
Source: Nat Nanotechnol. 2026 Jan 21;21(2):216–22. doi: 10.1038/s41565-025-02054-4 (PMC12916311; doi:10.1038/s41565-025-02054-4)
Supplement: Supplementary file 1 — Supplementary Figs. 1–19, Tables 1 and 2, Experimental methods, Sample preparation, Analysis of coupling regime, Dluence study, Simulation details, and Theoretical modelling and estimates. [file 41565_2025_2054_MOESM1_ESM.pdf]

nature nanotechnology

Supplementary information https://doi.org/10.1038/s41565-025-02054-4

Ultrafast transition from coherent to incoherent polariton nonlinearities in a hybrid 1L-WS_2/plasmon structure

In the format provided by the authors and unedited

Table of content

1. Sample preparation 2
2. Angle-resolved linear reflectivity 3
3. Lumerical FDTD Simulation 4
4. Estimate of the coupling regime 5
5. Fluence study 10
6. 2DES Experimental details 11
7. Density matrix simulations 12
8. Model validation and coupling strength estimation 20
   Estimation of the effective exciton momentum in the vicinity to the nanoslit array 20
   Coupling strength ratio 21
9. 3-COM for TMD coupled infinitely long metal nanorods 21
   Individual exciton and plasmon harmonic oscillator equations 21
   Emitted electric fields 22
   Coupled equations 23
   Final set of 3 coupled oscillator equations 24
10. Deviation from bosonic excitons - Pauli-blocking 25
11. Additional data 26
12. References 27

1. Sample preparation

Polycrystalline silver films with a thickness of 200 nm are deposited on a fused silica substrate using an e-beam physical vapor evaporation process. Ga-based focused ion beam milling is used to fabricate plasmonic nanoslit arrays with a size of 20 x 50 µm^2. The width and depth of the slits are set to 45 nm and the period of the slit is chosen as $a_0 = 495$ nm (Fig. S1b). The slit array is coated with a 5-nm-thick layer Al_2O_3 (grown at 150°C) to avoid hot electron transfer between Ag and 1L-TMD.[1-3]

1L-WS_2 flakes are prepared by micro-mechanical exfoliation from bulk 2H-WS_2 (HQ Graphene source) on Nitto Denko tape,[4] and then exfoliated again on a polydimethylsiloxane (PDMS) stamp placed on a glass slide for inspection under an optical microscope. Optical contrast is optimized to identify 1L-WS_2 before dry transfer.[5] Selected flakes are aligned and stamped onto the nanoslit array (Fig. S1a) with x,y,z micro-manipulators at 60°C, before increasing the temperature to 80°C, so the flakes detach from the PDMS and adhere preferentially to the substrate.

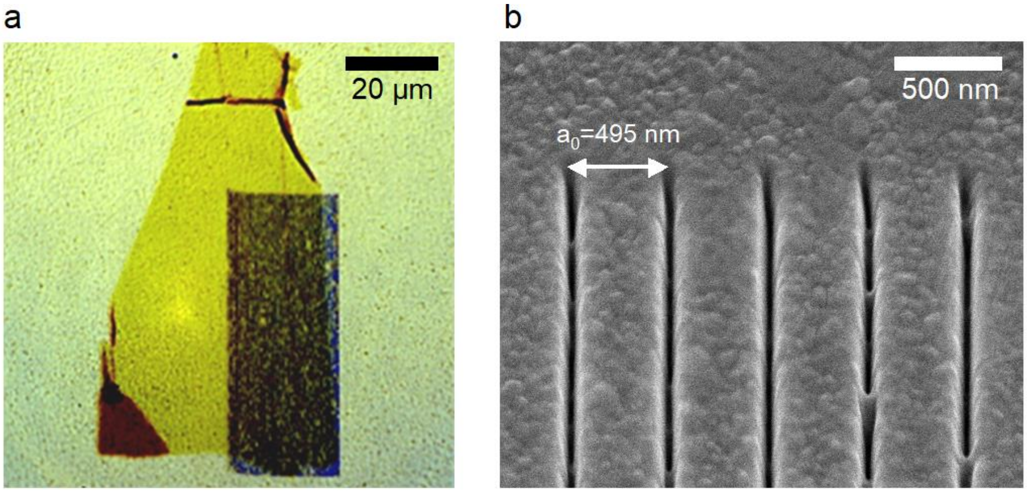

***Figure S1**: **a:** Optical micrograph of the sample, showing the grating and partially overlapping 1L-WS_2 flake. **b:** Scanning electron micrograph of the Ag nanoslit array with slit depth and width of 45 nm and a grating period of $a_0 = 495$ nm.*

Fig. S2 plots the Raman and photoluminescence (PL) spectrum of the transferred sample (Fig. S1a), respectively, taken onto the WS_2 area off-grid. The Raman spectrum (Fig. S2a) shows the main Raman modes at ~418.8 ± 0.2 cm^{-1} (A'_1), ~356.8 ± 0.2 cm^{-1} (E') and ~352.1 ± 0.2 cm^{-1} (2LAM(A)) and 8 additional Raman modes (labelled 1-8) that are expected for 1L-WS_2 under 514 nm laser excitation, as discussed in Ref. 6. The difference in Raman shift between the A'_1 and the E' peak of ~62 cm^{-1} is also consistent with 1L-WS_2.[6] Fig. S2b shows a PL emission at ~616.1 nm, also consistent with 1L-WS_2.[6]

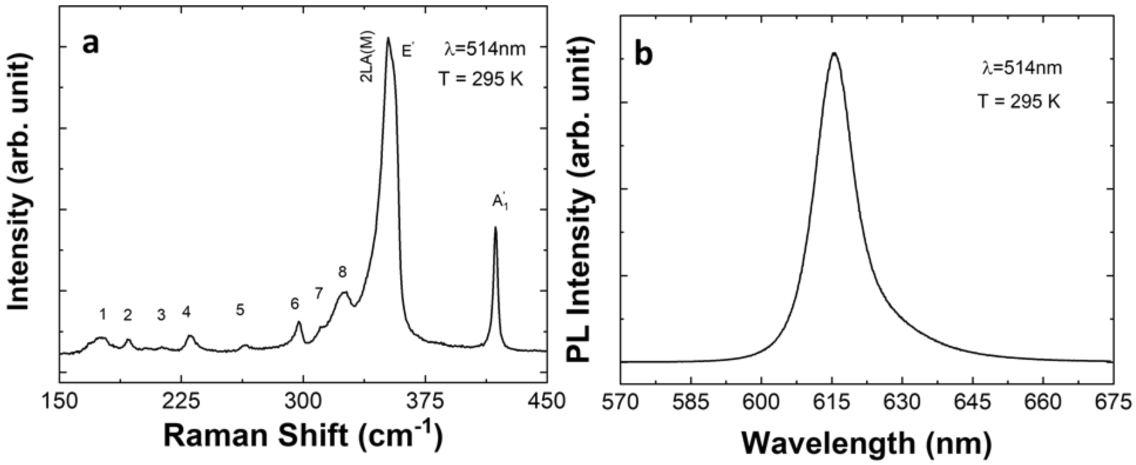

Figure S2: ***a:*** *Raman and* ***b:*** *PL spectra of 1L-*WS_2 *under 514 nm excitation at RT.*

2. Angle-resolved linear reflectivity

To characterize the Ag nanoslit arrays, we record angle-resolved linear reflectivity spectra before covering the nanoslit array with the 1L-TMD. For this we use an ultrabroadband white light source (Fianium WhiteLase micro), linearly polarized with a broadband Glan-Taylor polarizer (GT10), and focused onto the sample using a microscope objective (Nikon 10x E PLAN LWD, NA = 0.25) under normal incidence. The sample position in the focal plane of the objective is controlled by a 3d nano-positioning system. The light reflected from the sample is collected with the same microscope objective, separated from the incident light using a 50/50 beam splitter and focused into a spectrometer consisting of a spectrograph (PI Acton SP2500i) attached to a Peltier-cooled CCD camera (PI ProEM HS-512BX3). By using a cylindrical lens in front of the spectrograph, the spectrum of the reflected light is imaged along the horizontal axis of the attached CCD camera, while the vertical axis represents the Fourier plane containing the angle-dependent spectrum.

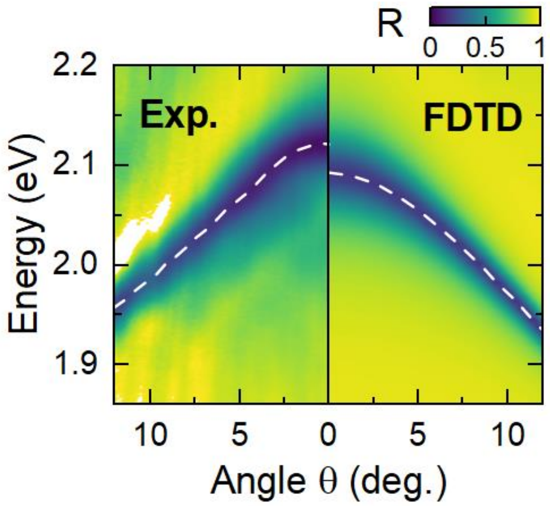

Figure S3: *Angle-resolved linear reflectivity spectra of a nanoslit array with a grating period of* a_0*=495 nm. Measurement (left) and FDTD simulation (right).*

Fig. S3 (left) shows the angle-dependent linear reflectivity of the nanoslit array for different angles θ, using the Al_2O_3 covered Ag film as a reference. The resonance energy follows the AM[-1] branch of the SPP dispersion relation.[7] A reduction in linewidth with increasing θ reflects the reduction in radiative SPP damping with decreasing SPP energy.[8] The angle-dependent linear reflectivity spectra of the nanoslit sample with the 1L-WS_2 are shown in Fig. 1b, measured using the same approach.

3. Lumerical FDTD Simulation

We perform Finite-Difference Time-Domain (FDTD) simulations of the angle-dependent linear reflectivity spectra and field distributions of Ag nanoslit arrays using the commercial Maxwell equation solver Lumerical FDTD Solutions. The geometrical parameters of the nanoslit arrays are taken from the scanning electron micrographs in Fig. S1.

Due to the translational invariance along the slits, it is sufficient to perform 2d simulations of the system in the $x - z$ −plane, where z is the surface normal and x is the axis perpendicular to the nanoslit array. To retrieve linear reflectivity spectra, a broadband plane-wave, covering the range from 1.7 to 2.3 eV, is injected into the simulation box, impinging onto the sample surface under a fixed angle θ with respect to the surface normal. A linear array of monitors is placed above the injection-plane of the incident plane wave. This monitor array records the reflected fields. Normalization to the incident field gives the sample reflectivity. Periodic boundary conditions are applied to the lateral boundaries, and perfectly matched layers are used at the top and bottom boundaries. To ensure θ-independence of the photon energy, the simulation is performed using the Broadband Fixed Angle Source Technique (BFAST).[9] The Ag dielectric function is taken from Ref. 10 and the dielectric function of Al_2O_3 is taken from Ref. 11. In the absence of 1L-WS_2, the resulting angle-dependent linear reflectivity spectra of the bare nanoslit array are in agreement with experiment (Figure S3). They reproduce both the angular dispersion and the linewidth of the SPP resonances of the array. Field distributions of the bare nanoslit array, taken at $\theta = 3°$ for the energy of the plasmon resonance (2.08 eV), are shown in Fig. S4. They show a pronounced enhancement of the x-component of the near field by about a factor of 20 in the slit region. The enhancement of the z-component is significantly weaker than the x-component of the near field, consistent with a previous study of a similar nanoslit array.[12] It is mainly this enhanced x-component inside the slits that is responsible for the coupling to the TMD excitons.

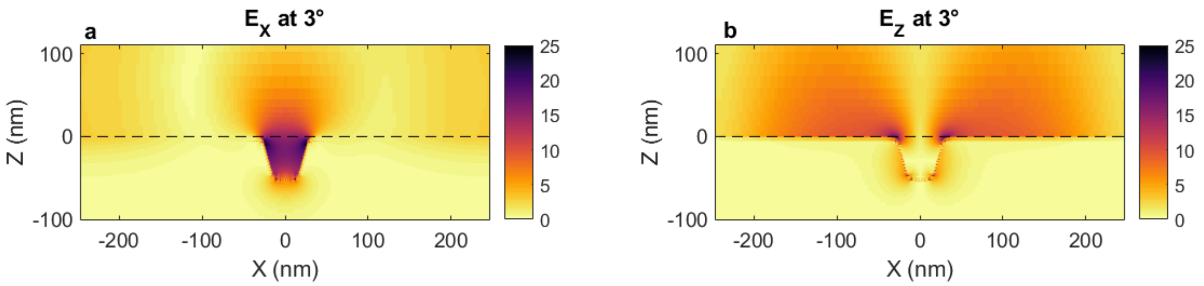

Figure S4: *Field distributions simulated using FDTD for the Ag nanoslit array covered with 5-nm Al_2O_3. The x- and z-components of the electric field (absolute values) are shown at the plasmon energy of 2.08 eV for plane wave excitation at $\theta = 3°$. Here, x denotes the direction parallel to the Al_2O_3-coated Ag surface, while z is the direction normal to the surface at z=0. One unit cell of the simulation with periodic boundary conditions is displayed. In this cell, the nanoslit is centered at x = 0.*

Similar simulations are carried out for a nanoslit array covered with 1L-WS_2. For this, we take an isotropic dielectric function for 1L-WS_2 with a frequency dependence given in Ref. 13. Very similar results are obtained when using an anisotropic dielectric function considering for the exciton resonance, taking only in-plane contributions into account. A good agreement between simulation and experiment can be observed in Fig. 1b. The comparable amplitude of UP and LP reflectivity dips is a signature that the oscillator strength of both polariton resonances is dominated by the plasmonic contribution to the wavefunction. This is visualized by comparing cross sections through the experimental and simulated linear reflectivity spectra at different angles (Fig. S5). When increasing θ, the wavefunction of the LP is mostly given by its plasmonic contribution, while that of UP becomes more exciton-like (see blue curves in Fig. S5). As such, the amplitude of the UP dip near 2.05 eV decreases strongly with increasing angle. From the data in Fig. S5 and the reflectivity spectra of 1L-WS_2,[14] we estimate a ratio of the oscillator strengths of plasmon and exciton of at least 25.

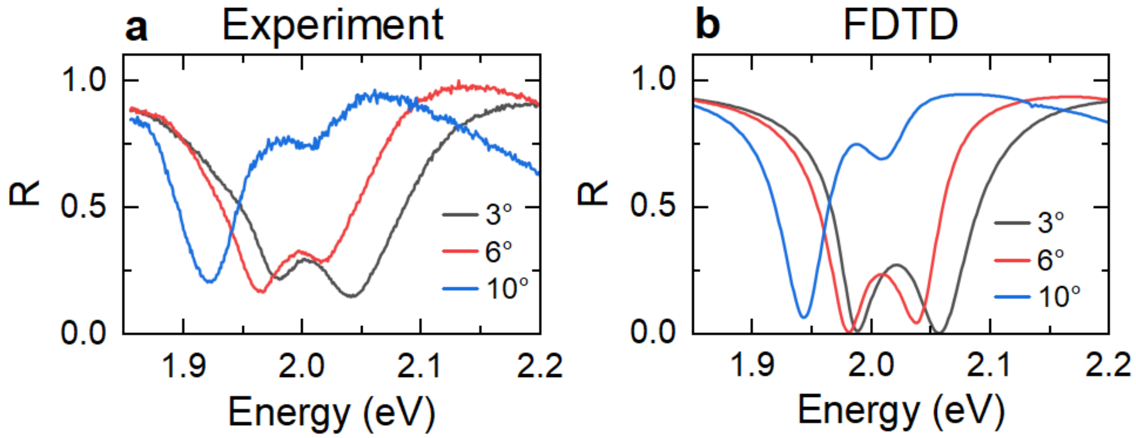

Figure S5: *Crosscuts through (a) experimental and (b) simulated linear dispersion for selected θ. Pump-probe and 2DES are performed at $\theta = 3°$ (black lines), slightly above the crossing at $\theta \approx 6°$ (red lines). While UP and LP amplitudes are comparable close to the crossing angle, for larger detunings ($\theta = 10°$, blue lines) UP and LP become more exciton- and plasmon-like, respectively. The different amplitudes highlight that the oscillator strength of the SPP is much larger than that of the exciton.*

4. Estimate of the coupling regime

While we will later use a coupled oscillator model (COM) that considers the coupling of bright and dark excitons to the SPP,[15, 16] we are restricting our analysis of the linear reflectivity spectra to a phenomenological, non-Hermitian two-oscillator model. This is justified, since in the parameter regime of our sample only UP and LP will significantly contribute to the linear spectra. The coupling strength V_{XP} resulting from this analysis will thus be an effective coupling strength.

We consider a bright exciton $|X\rangle$ with energy $E_X - \mathrm{i}\hbar\gamma_X$ and transition dipole moment μ_X. The dephasing of the exciton polarization is introduced via an imaginary part $\hbar\gamma_X$ of the exciton energy. The dephasing rate $\gamma_X = 1/T^*_{2,X}$ is related to the pure exciton dephasing time $T^*_{2,X}$. The exciton is coupled to a bright plasmon mode $|P\rangle$ with energy $E_P - i\hbar\gamma_P$ and transition dipole moment μ_P. A finite plasmon lifetime $T_{1,P}$ (decay rate $\kappa_P = 1/T_{1,P}$) gives $\gamma_P = \kappa_P/2$. Effects due to finite inhomogeneous broadening are neglected. For linear spectra, it is sufficient to treat these two excitations as two-level systems.

We obtain the (non-Hermitian) system Hamiltonian

$$\hat{H}_S = \begin{pmatrix} 0 & 0 & 0 \\ 0 & E_X - i\hbar\gamma_X & V_{XP} \\ 0 & V_{XP}^* & E_P - i\hbar\gamma_P \end{pmatrix}. \quad \text{(S1)}$$

We will further only consider real-valued coupling elements, i.e. $V_{XP}^* = V_{XP}$.

The transition dipole moment operator reads

$$\hat{\mu} = \begin{pmatrix} 0 & \mu_X & \mu_P \\ \mu_X^* & 0 & 0 \\ \mu_P^* & 0 & 0 \end{pmatrix}. \quad \text{(S2)}$$

Solving this Hamiltonian yields, in addition to the unaltered ground state $|0\rangle$, two new UP and LP eigenstates with wave functions

$$\begin{aligned} |UP\rangle &= c_{X,UP}|X\rangle - c_{P,UP}|P\rangle \\ |LP\rangle &= c_{X,LP}|X\rangle + c_{P,LP}|P\rangle = \sqrt{1 - c_{X,UP}^2}|X\rangle + \sqrt{1 - c_{P,UP}^2}|P\rangle \end{aligned} \quad \text{(S3)}$$

where the coefficients $c_{i,j}$ are the amplitudes of the $i = \{X, P\}$ contribution for the $j = \{UP, LP\}$. For negative coupling strengths, the LP is the in-phase superposition of X and P, while UP is the out-of-phase superposition. For positive coupling strengths, we arrive at the opposite scenario. Fig. S6 shows an exemplary polariton composition as a function of the detuning between X and P.

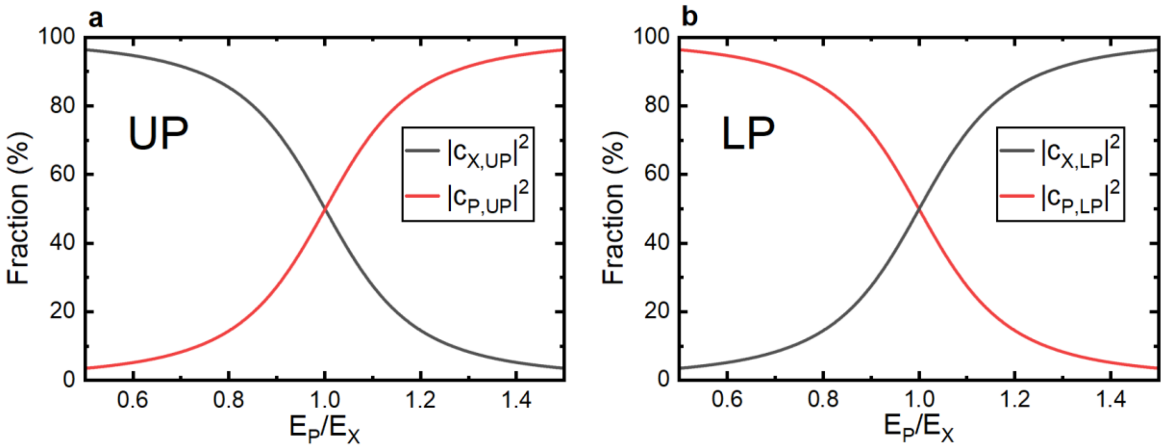

Figure S6: *Polariton composition for (a) UP and (b) LP in terms of their respective exciton ($|c_X|^2$) and plasmon ($|c_P|^2$) contributions for fixed E_X as a function of the plasmon detuning. A normalized coupling strength of $V_{XP} = 0.1E_X$ is used. For $E_X = E_P$ (the crossing), UP and LP contain equal contribution of X and P, although with different signs for their wavefunction amplitudes.*

We obtain the polariton eigenenergies

$$E_{UP} = \frac{E_X + E_P - i(\gamma_X + \gamma_P)}{2} + \frac{1}{2}\sqrt{4\left(-E_X E_P + V_{XP}^2 + \gamma_X\gamma_P + i(E_X\gamma_P + E_P\gamma_X)\right) + \left(E_X + E_P - i(\gamma_X + \gamma_P)\right)^2}$$
$$E_{LP} = \frac{E_X + E_P - i(\gamma_X + \gamma_P)}{2} - \frac{1}{2}\sqrt{4\left(-E_X E_P + V_{XP}^2 + \gamma_X\gamma_P + i(E_X\gamma_P + E_P\gamma_X)\right) + \left(E_X + E_P - i(\gamma_X + \gamma_P)\right)^2}. \quad \text{(S4)}$$

In the degenerate case, i.e. $E_X = E_P$, the energies of UP and LP simplify to

$$E_{UP} = E_X - \frac{i(\gamma_X + \gamma_P)}{2} + \frac{1}{2}\sqrt{4V_{XP}^2 - (\gamma_P - \gamma_X)^2}$$
$$E_{LP} = E_X - \frac{i(\gamma_X + \gamma_P)}{2} - \frac{1}{2}\sqrt{4V_{XP}^2 - (\gamma_P - \gamma_X)^2}. \quad \text{(S5)}$$

Here, the polariton energies remain degenerate even for finite coupling strengths as long as the term inside the square root stays negative, giving rise to the **weak coupling** regime[17, 18]

$$V_{XP} < V_{WC} = \sqrt{\frac{1}{4}(\gamma_X - \gamma_P)^2}, \quad \text{(S6)}$$

as is shown in Fig. S7. If $V_{XP} > V_{WC}$, we enter the **intermediate coupling** regime.[17, 18] This also marks the critical coupling strength for which the exciton and plasmon contribution of the polaritons are equal for both UP and LP, i.e. $c_X = c_P = 1/\sqrt{2}$ (Fig. S6), which is not the case for the weak coupling regime.

While a coupling strength V_{WC} is sufficient to create new polaritonic eigenstates with distinct new eigenenergies, characterized by an energy difference, the normal mode splitting (NMS) $E_{NMS} = E_{UP} - E_{LP}$, their finite spectral linewidth can make it hard to distinguish them. It is therefore helpful to define a minimal coupling strength for which UP and LP are sufficiently separated to resolve them as two individual resonances. This is the **strong coupling** regime[17, 18]

$$V_{XP} > V_{SC} = \sqrt{\frac{1}{2}(\gamma_X^2 + \gamma_P^2)}. \quad \text{(S7)}$$

where the coupling is stronger than the mean damping (Fig. S7).

The transition dipole moments of UP and LP are defined by μ_X and μ_P and their wavefunctions as

$$\mu_{UP} = c_X\mu_X - c_P\mu_P$$
$$\mu_{LP} = c_X\mu_X + c_P\mu_P. \quad \text{(S8)}$$

For $V_{XP} < 0$ and degenerate X and P, LP will have more oscillator strength $f_{LP} \propto |\mu_{LP}|^2$ than the UP.

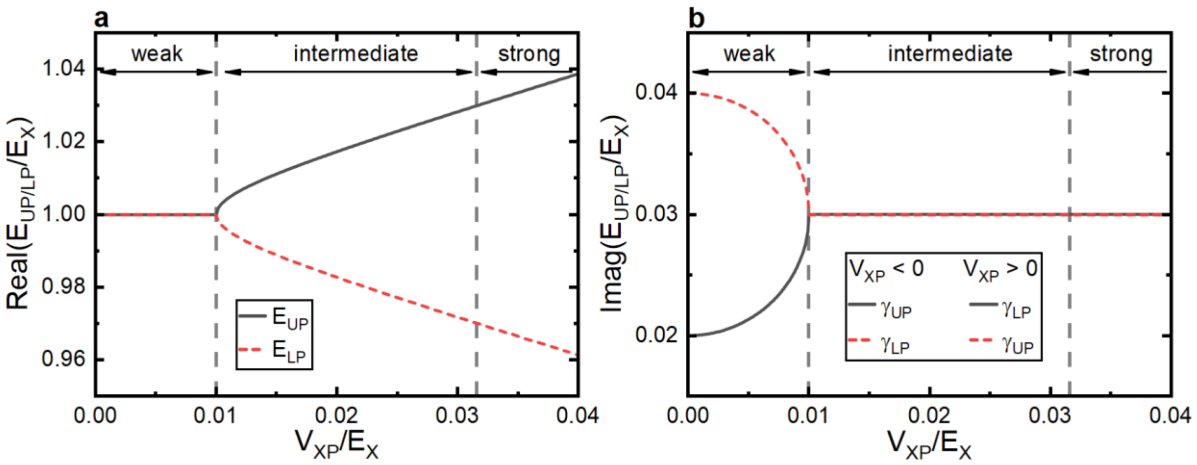

Figure S7: *Effect of coupling strength on polariton energies and damping in the degenerate case ($E_X = E_P$) using a normalized damping of $\gamma_X = 0.02E_X$ and $\gamma_P = 0.04E_X$.* ***a:*** *For the weak coupling regime UP and LP are degenerate. For the chosen dampings, the weak coupling regime ends at $V_{XP} = 0.01E_X$. Strong coupling begins at $V_{XP} \approx 0.032E_X$.* ***b:*** *Corresponding polariton dampings. In the weak coupling regime, the UP and LP dampings approach each other when increasing the coupling strength. For $V_{XP} > V_{WC}$, both UP and LP have the same damping and therefore spectral linewidth.*

Effects of bright and dark excitons on the polariton formation

To realistically model our experiments, it is necessary to account for SPP coupling to both momentum bright and dark excitons, $|X_B\rangle$ and $|X_D\rangle$.[15, 16] To analyze the effects of these exciton states on the polariton energies and wavefunctions, we neglect any damping.

Our Hamiltonian reads

$$\widehat{H}_S = \begin{pmatrix} 0 & 0 & 0 & 0 \\ 0 & E_{X_B} & V_{X_BP} & 0 \\ 0 & V_{X_BP}^* & E_P & V_{X_DP} \\ 0 & 0 & V_{X_DP}^* & E_{X_D} \end{pmatrix} \tag{S9}$$

with transition dipole moment operator

$$\hat{\mu} = \begin{pmatrix} 0 & \mu_{X_B} & \mu_P & \mu_{X_D} \\ \mu_{X_B}^* & 0 & 0 & 0 \\ \mu_P^* & 0 & 0 & 0 \\ \mu_{X_D}^* & 0 & 0 & 0 \end{pmatrix}, \tag{S10}$$

with $\mu_{X_D} = 0$. When choosing the degenerate case, i.e. $E_{X_D} = E_{X_B} = E_P$, we obtain 3 eigenstates, upper and lower polaritons $|UP\rangle$ and $|LP\rangle$ and a third state, which we refer to as a "dark" state $|D\rangle$. Their eigenenergies are

$$E_{UP} = E_X + \sqrt{V_{X_BP}^2 + V_{X_DP}^2}$$
$$E_D = E_X \qquad \text{(S11)}$$
$$E_{LP} = E_X - \sqrt{V_{X_BP}^2 + V_{X_DP}^2}.$$

For $V_{X_BP} = V_{X_DP}$, the effective coupling strength $V_{XP} = \sqrt{V_{X_BP}^2 + V_{X_DP}^2}$, is enhanced by a factor of $\sqrt{2}$ over that achieved with one exciton. This equals the $N = 2$ emitter limit of the Tavis-Cummings (TC) Hamiltonian.[19, 20] The new "dark" state $|D\rangle$ that arises in the presence of for two excitons, sits at the exciton energy. This is even the case when the plasmon is detuned from the exciton.

The wavefunction of these three states, in the degenerate case, are of the form

$$|UP\rangle = c_{X,UP}|X_B\rangle + c_{X,UP}|X_D\rangle - c_{P,UP}|P\rangle$$
$$|D\rangle = \mathrm{c}_{\mathrm{X_B},D}|X_B\rangle - \mathrm{c}_{\mathrm{X_D},D}|X_D\rangle \qquad \text{(S12)}$$
$$|LP\rangle = c_{X,UP}|X_B\rangle + c_{X,UP}|X_D\rangle + c_{P,UP}|P\rangle.$$

Therefore, both excitons contribute to UP and LP with the same amplitude. The $|D\rangle$ state is purely excitonic and does not contain any plasmon. In particular for the scenario where $V_{X_BP} = V_{X_DP}$, we obtain

$$|UP\rangle = \frac{1}{2}|X_B\rangle + \frac{1}{2}|X_D\rangle - \frac{1}{\sqrt{2}}|P\rangle$$
$$|D\rangle = \frac{1}{\sqrt{2}}|X_B\rangle - \frac{1}{\sqrt{2}}|X_D\rangle \qquad \text{(S13)}$$
$$|LP\rangle = \frac{1}{2}|X_B\rangle + \frac{1}{2}|X_D\rangle + \frac{1}{\sqrt{2}}|P\rangle$$

which are again the wavefunctions for the $N = 2$ TC Hamiltonian.[21]

As before, the transition dipole moments of the eigenstates are given by their wavefunctions. UP and LP therefore are both gaining transition dipole moment from both excitons, while the "dark" state D would be fully dark in case of $\mu_{X_B} = \mu_{X_D}$ (TC case). For our coupling scenario ($\mu_{X_D} = 0$) the transition dipole moment of the dark state is $\mu_D = \mu_{X_B}/\sqrt{2}$, while the plasmon transition dipole moment is contributing to both, UP and LP. For $\mu_P \gg \mu_{X_B}$, D may therefore be classified as a "grey" state.

Coupling strength

To quantitatively determine the coupling regime for the 1L-WS_2/plasmonic nanostructure, we analyze the angle-dependent linear reflectivity spectra (Fig. S8a) using a Lorentz oscillator model. The deduced NMS between the UP and LP is shown in Fig. S8b as a function of θ. Minima in the NMS mark the crossing angle $\theta_c \approx \pm 6°$ between X and SPP. To determine the coupling regime, we need to compare this value to the damping of the uncoupled systems. For this, we deduce $\hbar\gamma_X$ and $\hbar\gamma_P$ (half width at half maximum) from the angle-dependent resonances of the SPP (Fig. S3) and 1L-WS_2 on the bare Ag substrate, Fig. S8b. We then determine the coupling strength for reaching the strong coupling[17, 18] limit using Eq. (S7), blue line in Fig. S8b. In the crossing, the NMS corresponds to $E_{NMS} = 2V_{XP}$.The analysis shows that the sample is at

the border between intermediate and strong coupling regimes, depending on θ. In particular, for $\theta = 6°$ we get a NMS of 48 meV, $\hbar\gamma_X = 19$ meV and $\hbar\gamma_P = 34$ meV. This yields a coupling strength of $V_{XP} \approx 24$ meV. For these values, the strong coupling limit $V_{SC} = 27.5$ meV and would therefore require a NMS of 55 meV, ~15% higher than that seen in experiment.

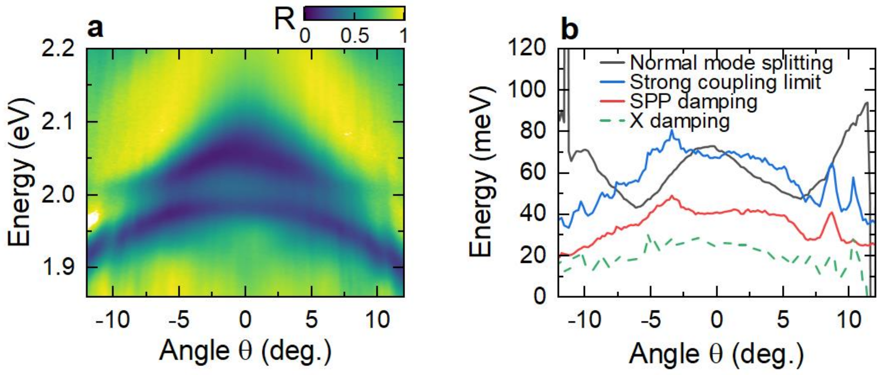

Figure S8: *Analysis of the coupling regime for 1L-WS_2/plasmonic nanostructure.* ***a:*** *Experimental angle-dependent linear reflectivity spectra of the hybridized system, including negative θ.* ***b:*** *Results of a Lorentz oscillator analysis of linear reflectivity spectra, from which the normal mode splitting E_{NMS} is deduced (black line). Exciton (green dashed line) and SPP (red line) damping (half width at half maximum) are obtained from the uncoupled constituent systems (see Fig. S3 for the SPP). From these, the normal mode splitting that marks the strong coupling limit is computed (blue line). A minimum of E_{NMS} marks the crossing angles $\theta_c \approx \pm 6°$.*

5. Fluence study

In order to avoid fluence-dependent signal contributions, in particular those that arise from nonlinear signals that go beyond third order perturbation theory, we study the fluence dependence of the $\Delta R/R$ spectra of the 1L-WS_2-covered nanostructure. For this, we use collinearly *p*-polarized pump and probe pulses, with the E-field vector set perpendicular to the grating lines. The $\Delta R/R$ spectra are recorded for $\theta = 6°$. The pump fluence is varied by two orders of magnitude from 1 to 100 µJ/cm². The probe is set to 20 µJ/cm². We ensured that the probe fluence itself does not induce observable changes in the lineshape of the linear reflectivity. Spectral crosscuts at $T = 100$ fs are displayed in Fig. S9a. These data show for higher fluences slight spectral lineshape changes at the low-energy side of the exciton resonance. Since the changes mainly affect the lineshape in the LP, but not in the UP region, it is likely that they are not directly associated with the polariton resonances. The spectral changes are accompanied by changes in the early dynamics, as highlighted in Fig. S9b at the minimum of the differential signal at $E_{det} = 1.98$ eV. The pump-fluence dependence of the absolute signal strength, taken from the average of the data in Fig. S9b for $T > 0$, are presented in Fig. S9c. A saturation effect is seen in the differential reflectivity for high fluences starting above ~10 µJ/cm². A linear fit confirms that the pump fluence of 10 µJ/cm², used in the experiments, is sufficiently low and that the recorded signal is in the linear regime of third order nonlinearities.

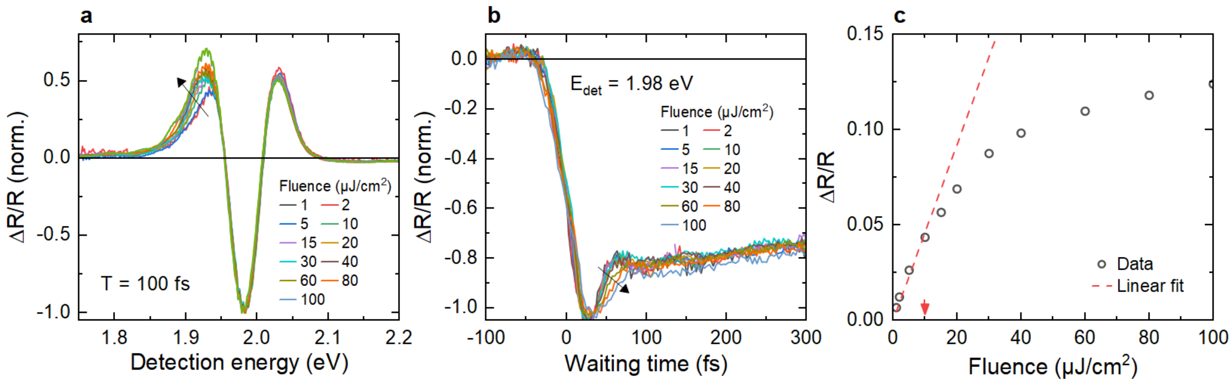

Figure S9: *Fluence dependence of $\Delta R/R$ spectra for 1L-WS_2 on the Ag nanoslit array.* ***a:*** *Spectral crosscut at $T = 100$ fs for fluences from 1 to 100 μJ/cm². **b:** Dynamics taken at $E_{det} = 1.98$ eV for the same fluences. **c:** Absolute value of the $\Delta R/R$ signal as function of fluence, taken as the average signal at $E_{det} = 1.98$ eV for $T > 0$. Experiments are performed at 10 μJ/cm².*

6. 2DES Experimental details

Ultrafast pump-probe and 2DES experiments are conducted using a home-built noncollinear optical parametric amplifier (NOPA) system and 2DES setup, previously described in Ref. 14. A fiber amplifier system (Tangerine V2, Amplitude Systems) delivers 260-fs pulses (FWHM of the intensity profile) centered at 1030 nm at 175 kHz repetition rate. A fraction with ~50 μJ pulse energy is used to pump a NOPA that utilizes third harmonic generation for pumping and is based on a design introduced in Ref. 22. The output spectrum of the NOPA and the spectral stability in Fig. S10b are measured with a fast line camera (Aviiva EM4, e2v) at half of the laser repetition rate, i.e. 87.5 kHz, for 10000 consecutive spectra. A second harmonic frequency-resolved optical gating (SH-FROG) measurement of the cross-correlation between the pump and probe beam in the 2DES setup at the sample position using a 10-μm β-BBO crystal is shown in Fig. S10a, yielding a retrieved pulse duration of ~9 fs.

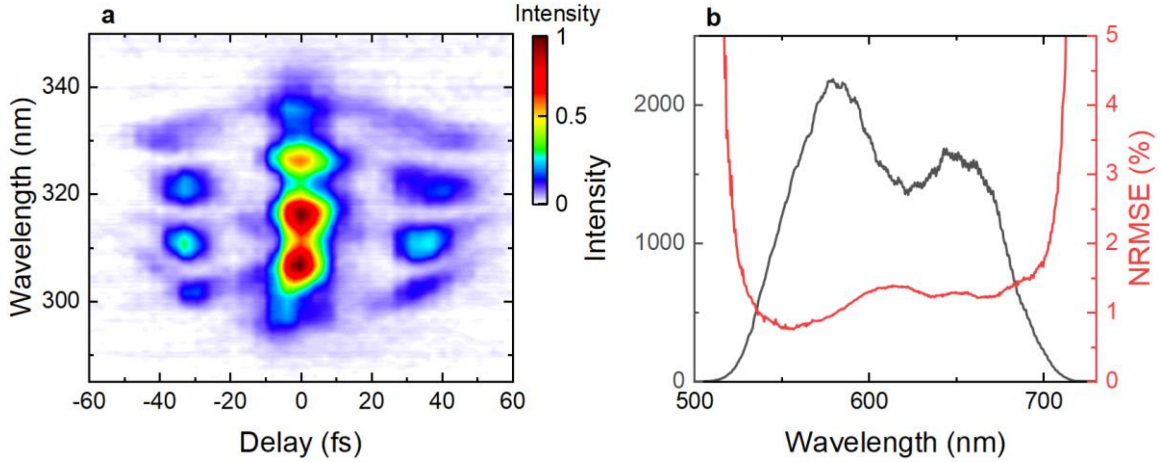

Figure S10: ***a:*** *Cross-correlation second harmonic frequency-resolved optical gating measurement between pump and probe at the sample position using a 10-μm β-BBO crystal. The retrieved pulse duration is ~9 fs.* ***b:*** *Laser spectrum and stability as normalized root-mean square error of 10000 spectra at 87.5 kHz.*

The 2DES setup splits the NOPA beam into a pump and a probe. The pump is fed into a common-path birefringent interferometer (Translating Wedge-based Identical pulse eNcoding System, TWINS)[23] to create the phase-stable excitation pulse pair with delay τ. A relative delay between the pump and probe (waiting time T) is set by using a motorized retroreflector (M126.DG, Physik Instrumente) for the probe beam before both beams are focused onto the sample using an off-axis parabolic mirror (50 mm reflected focal length). The pump and probe are focused to ~19x19 µm² and ~17x12 µm², respectively. The reflected beams are re-collimated using the same mirror and sent to a grating spectrograph (Acton SP2150i, Princeton Instruments) with an attached fast line camera (Aviiva EM4, e2v), operating at half of the laser repetition rate (87.5 kHz). The pump is chopped in pairs of two consecutive pulses at a rate of 43.75 kHz using a synchronized rotating chopper (MC2000B, Thorlabs) with a custom-made 500 slot wheel. Spectra S of the probe laser light reflected from the sample are recorded with the pump being either switched on (S_{on}) or off (S_{off}). From these, different reflectivity spectra

$$\frac{\Delta R}{R}(\tau, T, E_{det}) = \frac{S_{on}(\tau, T, E_{det}) - S_{off}(E_{det})}{S_{off}(E_{det})} \tag{S14}$$

are computed as a function of the coherence time τ, waiting time T and detection energy E_{det}. In pump-probe, T is varied while we keep $\tau = 0$. For 2DES, τ is scanned from -50 to 160 fs for each T, and differential spectra are recorded on the fly. Simultaneously, a field autocorrelation of the excitation pulse pair is recorded using a photo diode.[24, 25] This measurement is used to determine $\tau = 0$ and for phase correction of the data. 2DES maps are calculated from a coherence time scan via Fourier transform

$$A_{2D}(E_{ex}, T, E_{det}) = \Re\left(\int_{-\infty}^{\infty} \Theta(\tau) \frac{\Delta R}{R}(\tau, T, E_{det}) e^{iE_{ex}\tau/\hbar} \mathrm{d}\tau\right). \tag{S15}$$

along τ, yielding absorptive energy-energy maps as a function of the detection energy E_{det} and excitation energy E_{ex}. Here, $\Theta(\tau)$ is the Heaviside step function. The excitation energy axis is obtained after Fourier transformation via a previously recorded calibration scan to account for the spectral dependence of the birefringence used by the TWINS to create the excitation pulse pair.[24, 25]

7. Density matrix simulations

Simulations of linear spectra, density matrix dynamics and 2DES signals are carried out using a non-perturbative approach based on numerically solving the Lindblad master equation of the system while accounting for all time-dependent field interactions.[12, 14] Ref. 14 provides details about the implementation of these simulations.

We numerically solve the master equation in Lindblad form,[26, 27] accounting for all 3 laser electric fields using Gaussian-shaped laser pulses with a FWHM of the intensity profile of 5 fs. The pulses are centered at 2.15 eV. Their amplitude is chosen to be sufficiently weak such that the interaction energy $\mu \cdot E$, with transition dipole moment μ and electric field amplitude E, is much weaker than the electronic coupling elements of the system Hamiltonian $\widehat{H}_S$. To simulate the experiments, the optical response is obtained by calculating the expectation value of the transition dipole moment operator along the detection time for fixed τ and T. Using a 4-step phase-cycling scheme, the nonlinear third-order polarization is then isolated from the total signal and 2DES maps are obtained by varying τ and T accordingly.

System Hamiltonian and Lindblad Terms

In the simulations, we use a coupled oscillator model (COM) comprising two exciton resonances, a bright exciton (X_B) and a dark exciton (X_D). Both excitons are coupled to a plasmonic mode P. The idea behind this approach is to follow a minimal oscillator model proposed for hybrid TMD plasmon systems.[15, 16] Details of the exciton fine structure in 1L-WS_2, specifically the valley degree of freedom[28] and spin-orbit couplings resulting in the formation of A and B excitons,[28] are neglected in order to focus on the central aspects of the A exciton-plasmon coupling and the resulting polariton formation in 1L-WS_2. We emphasize the phenomenological character of this COM, intended to assign the physical origin of the nonlinearity of the coupled system.

Using these two kinds of excitons $X = \{X_D, X_B\}$, we set up the system Hamiltonian as[12]

$$\hat{H}_S = E_P \hat{b}_P^\dagger \hat{b}_P + \sum_X E_X \hat{b}_X^\dagger \hat{b}_X + V_{XP}\left(\hat{b}_X^\dagger \hat{b}_P + \hat{b}_P^\dagger \hat{b}_X\right) \tag{S16}$$

by employing exciton creation and annihilation operators $\hat{b}_X^\dagger$ and $\hat{b}_X$, respectively, with A exciton energy $E_X = 2$ eV used for both momentum bright and dark excitons X_B and X_D. For the plasmon, we use the operators $\hat{b}_P^\dagger$ and $\hat{b}_P$ and plasmon energy E_P, which varies approximately linearly with θ. Interactions between the excitons and the plasmon are accounted for by the light matter coupling in rotating wave approximation[12] with coupling strengths V_{XP}. We describe the coupling of excitons and plasmons to the external laser pulses by introducing the transition dipole moment operators for X and P as[12]

$$\hat{\mu}_{X/P} = \mu_{X/P}\left(\hat{b}_{X/P}^\dagger + \hat{b}_{X/P}\right). \tag{S17}$$

The radiative damping of the plasmon is considered using a Lindblad operator[12]

$$\hat{L}_{rel,P} = \sqrt{\kappa_P}\hat{b}_P \tag{S18}$$

with plasmon lifetime $T_{1,P} = 1/\kappa_P$. Similarly, we introduce exciton dephasing via[12]

$$\hat{L}_{dep,X} = \sqrt{2\gamma_X}\hat{b}_X^\dagger \hat{b}_X, \tag{S19}$$

using two independent baths for X_D and X_B.

X-P nonlinearity

Excitation-induced dephasing: So far, the employed Hamiltonian consists of a ground state $|0\rangle$, 3 one-quantum (1Q) states comprising the 2 excitons $|X_D\rangle$ and $|X_B\rangle$ and the plasmon $|P\rangle$ and 6 two-quantum (2Q) states. The 2Q states include two-exciton excitations $|XX_D\rangle$ and $|XX_B\rangle$, a mixed two-exciton state $|X_B, X_D\rangle$, the two-plasmon state $|2P\rangle$ and the mixed X-P states $|X_D, P\rangle$ and $|X_B, P\rangle$. As long as the model is restricted to purely harmonic quantum-mechanical oscillators, no optical nonlinearity arises. This holds even for finite V_{XP}. In good agreement with earlier experimental results,[12, 29, 30] we consider the plasmonic excitations of the nanoslit array as purely harmonic oscillators, neglecting any nonlinearity of the structured metal film which is orders of magnitude weaker than the relevant excitonic nonlinearities[31] (see also Fig. S18). The excitonic excitations, however, exhibit a strong nonlinear optical response (Fig. S11). As

demonstrated in previous work,[14, 32] these nonlinearities arise, for 1L-TMD excitons, predominantly from many-body interactions (MBIs) such as excitation-induced dephasing (EID).[33] In our model simulations, we include EID for the coupled system by modification of the excitonic Lindblad operator, i.e., by increasing the linewidth of the $|X\rangle \to |XX\rangle$ transitions relative to that of the $|0\rangle \to |X\rangle$ transition.[14] In the simulations we assume a 10% increase in the linewidth ($\gamma'_X = 1.1\gamma_X$), both for X_D and X_B.[14] Via coupling to the plasmon, this MBI is transferred to the polaritons, resulting in a finite optical nonlinearity of both LP and UP.[34]

Pauli blocking: Another possible nonlinearity of the excitonic system arises from phase-space filling, i.e., Pauli blocking. This nonlinearity reduces the oscillator strength of the exciton after optical excitation (see also section 10).[33, 35] As a result, the strength of the X-P coupling in the 2Q manifold is reduced, leading to a reduction in the normal mode splitting of the polariton system.[29] This nonlinearity is usually referred to as a "Rabi contraction", i.e., reduction in normal mode splitting between LP and UP.[21, 36-38] In our simulations, we account for this phase-space filling and the resulting "Rabi contraction" by slightly reducing the $|X\rangle \to |XX\rangle$ transition dipole moment $\mu_{X,XX} = 0.99\sqrt{2}\mu_{0,X}$. A consequence of this is to introduce two origins for a nonlinearity: i) breaking the balance between GSB/SE and ESA pathways via their amplitude and ii) changing the energies of the 2Q states via Rabi contractions. The terms affected by this are the coupling elements between $|XX_D\rangle$ and $|X_D, P\rangle$ and between $|XX_B\rangle$ and $|X_B, P\rangle$. These elements are reduced to $0.99 \cdot V_{XP}$, leading to changes in the energies of $|2UP\rangle$ and $|2LP\rangle$ as well as of $|DUP\rangle$ and $|DLP\rangle$. Diagonalization of $\hat{H}_S$ shows that Pauli blocking increases (decreases) the energy of $|2LP\rangle$ ($|2UP\rangle$) by ΔE, while it increases (decreases) the energy of $|DLP\rangle$ ($|DUP\rangle$) by $\Delta E'$. The energy of $|2D\rangle$ is not altered and also not that of $|UPLP\rangle$ in the degenerate case (see Fig. 4a).

Excitation-induced shifts: In our phenomenological COM, the excitation of two-exciton states may in principle also affect the energy of the excitonic transition and result in a spectral lineshift of the two-exciton relative to the exciton transition.[12] Such EIS effects are indeed important for a quantitative understanding of the optical nonlinearities of 1L-WS_2.[14, 32, 33, 35, 39] In general, they give rise to dispersive line shapes of $\Delta R/R$ near the exciton resonance (see Fig. S11). We ignore EIS in our simulations for the nanoslit array covered with 1L-WS_2 since the experimental data in Figs. 2 and 3 did not show pronounced signs of such effects.

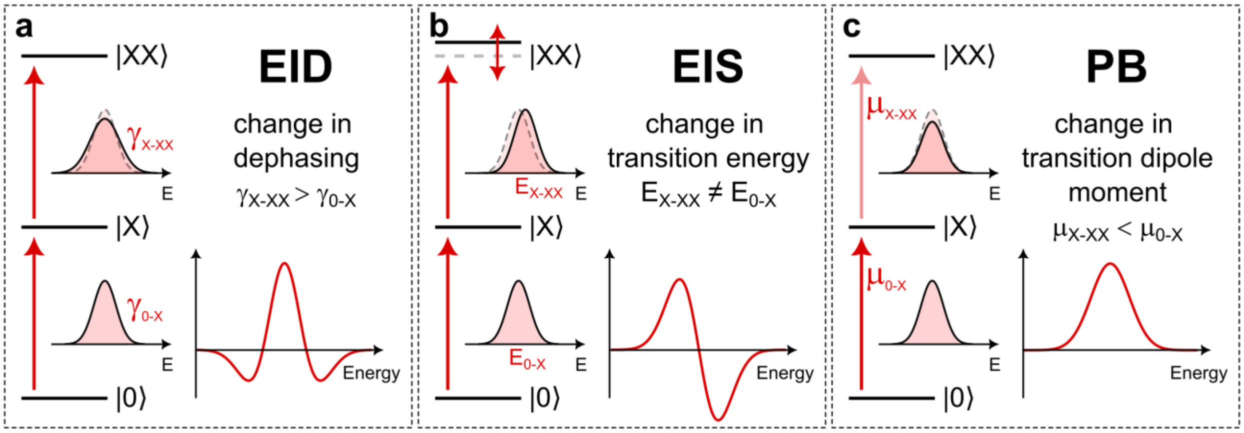

Figure S11: *Different nonlinearities for semiconductor excitons due to MBIs.* ***a:*** *EID leads to an increased damping γ of the $|X\rangle \to |XX\rangle$ transition relative to $|0\rangle \to |X\rangle$, resulting in characteristic line shapes with a*

positive central peak, reflecting a bleaching of the $|0\rangle \rightarrow |X\rangle$ transition, and negative side lobes arising from ESA due to the $|X\rangle \rightarrow |XX\rangle$ transition. ***b:*** *EIS energetically shifts the $|XX\rangle$ state, resulting in derivative line shapes of the nonlinear signal.* ***c:*** *Pauli blocking (PB) reduces the transition dipole moment μ for the $|X\rangle \rightarrow |XX\rangle$ transition relative to $|0\rangle \rightarrow |X\rangle$, resulting in a net positive bleaching-type signal.*

Hamiltonian in the uncoupled exciton-plasmon basis: In the uncoupled X-P basis, considering only a Rabi contraction nonlinearity, the sum of $\widehat{H}_S$ and light-matter interaction can be written up to the 2Q manifold:

$$\hat{H} = \begin{pmatrix} 0 & -\mu_{X_B}E(t) & 0 & -\mu_P E(t) & 0 & 0 & 0 & 0 & 0 & 0 \\ -\mu_{X_B}E(t) & E_{X_B} & 0 & V_{X_BP} & -0.99\sqrt{2}\mu_{X_B}E(t) & 0 & 0 & -\mu_P E(t) & 0 & 0 \\ 0 & 0 & E_{X_D} & V_{X_DP} & 0 & 0 & 0 & 0 & -\mu_P E(t) & -\mu_{X_B}E(t) \\ -\mu_P E(t) & V_{X_BP} & V_{X_DP} & E_P & 0 & 0 & -\mu_P\sqrt{2}E(t) & -\mu_{X_B}E(t) & 0 & 0 \\ 0 & -0.99\sqrt{2}\mu_{X_B}E(t) & 0 & 0 & 2E_{X_B} & 0 & 0 & 0.99\sqrt{2}V_{X_BP} & 0 & 0 \\ 0 & 0 & 0 & 0 & 0 & 2E_{X_D} & 0 & 0 & 0.99\sqrt{2}V_{X_DP} & 0 \\ 0 & 0 & 0 & -\mu_P\sqrt{2}E(t) & 0 & 0 & 2E_P & \sqrt{2}V_{X_BP} & \sqrt{2}V_{X_DP} & 0 \\ 0 & -\mu_P E(t) & 0 & -\mu_{X_B}E(t) & 0.99\sqrt{2}V_{X_BP} & 0 & \sqrt{2}V_{X_BP} & E_{X_B}+E_P & 0 & V_{X_DP} \\ 0 & 0 & -\mu_P E(t) & 0 & 0 & 0.99\sqrt{2}V_{X_DP} & \sqrt{2}V_{X_DP} & 0 & E_{X_D}+E_P & V_{X_BP} \\ 0 & 0 & -\mu_{X_B}E(t) & 0 & 0 & 0 & 0 & V_{X_DP} & V_{X_BP} & E_{X_B}+E_{X_D} \end{pmatrix}$$

(S20)

Diagonalization of the system Hamiltonian gives the eigenenergies of the 1Q and 2Q states in the polariton basis (Fig. 4a) while the transition dipole moments in the polariton basis are deduced from the eigenvectors of $\widehat{H}_S$ and the exciton and plasmon transition dipole moments.

Simulation parameters

The parameters used in the simulations are listed in Table S1, chosen to model the experimental data recorded for an angle of $\theta = 3°$, above the crossing between X and P, i.e. $E_P > E_X$, but with small detuning. Additional effects due to inhomogeneous broadening present in the investigated sample[14] are not accounted for in the simulations.

Table S1*: Parameters used in the simulations.*

Parameter	Value
E_{X_D}, E_{X_B}	2 eV
E_P	2.015 eV
V_{X_DP}	-19 meV
V_{X_BP}	-19 meV
$T_{1,P}$	12 fs
T^*_{2,X_D}, T^*_{2,X_B}	40 fs
μ_{X_D}	0
μ_{X_B}	0.1
μ_P	1

Similarities with a Tavis-Cummings Model

Effects of Rabi contractions on nonlinear optical spectra are often discussed as a nonlinearity for molecular polaritons[20, 21] in the framework of a Tavis-Cummings (TC) model.[40] Here, they arise since a finite number N of emitters is coupled to the cavity mode. In the 1Q manifold, this results in bright $|UP\rangle$ and $|LP\rangle$ states and $N-1$ dark states $|D\rangle$. In the limit of $N \to \infty$, and in the absence of MBI, the TC Hamiltonian becomes identical to a system of linear harmonic oscillators and the system will not display an optical nonlinearity.[19] We therefore expect that, when including a Pauli blocking nonlinearity, the TC Hamiltonian and our COM will show similar 2DES spectra for $T = 0$ fs. At early waiting times, dark states are not yet populated and therefore play a marginal role for the optical nonlinearity.

To test this assumption, we implement, as a first step, a TC model for $N = 2$ in the strong coupling regime, following Ref. 20 for the simulation parameters. The results and simulation parameters are shown in Fig. S12 and reproduce the results shown in Ref. 20 for $T = 0$ fs and $N = 2$. The 2DES map shows positive cross peaks and dispersive diagonal peaks as markers for coherent polaritons and a Rabi contraction nonlinearity.

Fig. S13 shows the simulation based on our COM using a single bright exciton coupled to the photonic state with the same parameters as for the TC simulation. The results are very similar to those of the TC Hamiltonian, in particular, we also obtain dispersive diagonal peaks and positive cross peaks. Minor differences can be seen in the line shapes, in particular for the ESA contributions. This is not surprising, since the COM with small Pauli blocking nonlinearity should be equivalent to the large-emitter-number limit of the TC model but different from the TC model for a small number of emitters.

This comparison shows that even though the microscopic electronic systems that are described by the COM and the TC models are quite different (a semiconductor system with delocalized exciton in the COM vs. a system of N identical molecular two-level emitters in the TC model), the expected nonlinear 2DES spectra are quite similar. This is the case since the Rabi contraction nonlinearity, important in both cases, can arise from two different microscopic origins: 1) phase-space filling (Pauli-blocking) for excitons of 1L-TMDs[36, 38] (see section 10) 2) finite number of emitters that is coupled to a cavity mode in the case of a molecular system.[20, 21]

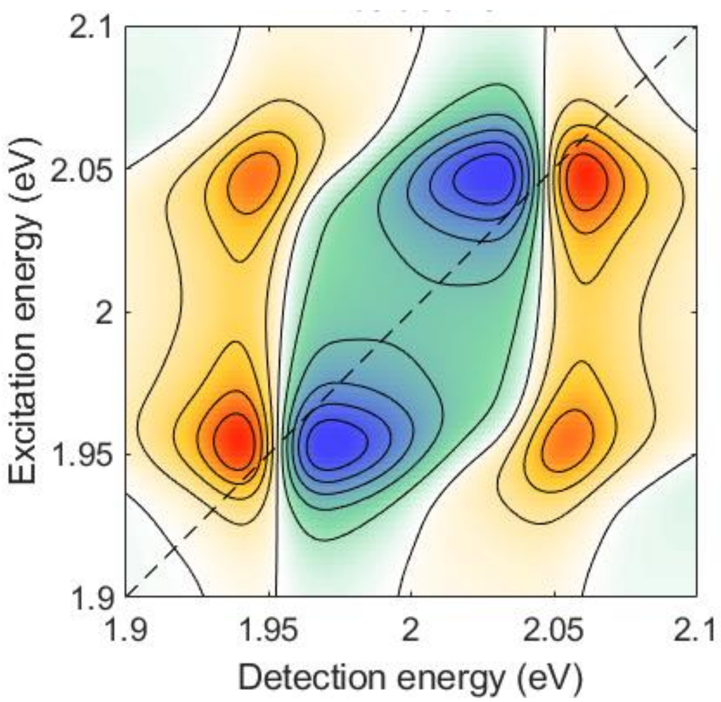

Figure S12: *2DES map for $T = 0$ fs for a TC Hamiltonian with $N = 2$. The following model parameters are used $E_P = E_X = 2$ eV, $\sqrt{N}\,V = 50$ meV, $T_{1,P} = 15$ fs, and $T_{2,X} = 50$ fs. We have chosen independent baths for all X and the transition dipole moments are taken as $\mu_X = 0$ and $\mu_P = 1$.*

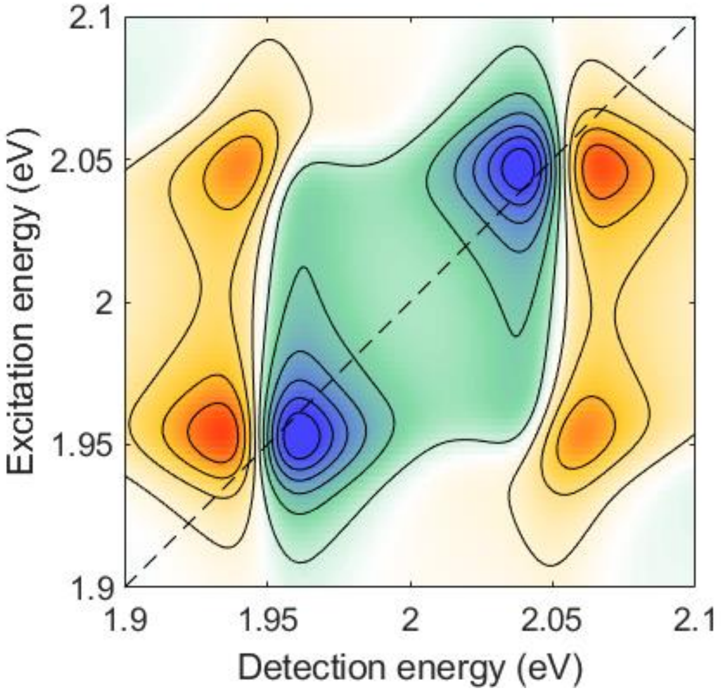

Figure S13: *2DES map at $T = 0\ fs$ for a COM with $N = 1$ exciton coupled to a plasmon. Identical model parameters as in the simulations for the TC model in Fig. S12, except for using $\mu_X = 0.01\ \mu_P$ and including Pauli blocking as the source for the nonlinearity with a factor of $\mu_{X,XX} = 0.99\sqrt{2}\,\mu_{0,X}$.*

Effects of dark states

To verify that the inclusion of dark states in the COM is indeed necessary for the description of the experimental 2DES maps, we perform comparative COM simulations in which we consider only a single bright exciton coupled to the plasmonic mode. The model parameters are the same as used in the main text. Fig. S14a shows that the linear spectrum remains unaffected by the absence of dark excitons. This can be understood based on the structure of the polariton wavefunctions discussed in section 4. In the parameter regime of our sample, the plasmon carries substantially more oscillator strength than the bright exciton. The polariton oscillator strengths are therefore mostly given by their plasmon component. Since the dark state has no plasmon contribution, its oscillator strength is limited to the exciton oscillator strength, hence it cannot be seen in the linear spectra in-between the UP and LP resonances.

Strong differences are, however, seen in the population dynamics (Figs. S14b,c 4b,c). Without the dark state $|D\rangle$, the polaritons are much shorter-lived (Fig. S14c). They decay with a lifetime of ~ 35 fs, given by the radiative decay of the plasmon mode. For times beyond ~100 fs almost no population is left. This is in contrast to experiment (Fig. 2), where we see a decay of the nonlinear signal with >10 ps.

2DES maps for $T = 0$ fs and 100 fs are shown in Fig. S15. They show essentially identical line shapes as in Fig. 4, even in the absence of dark states. Not only at early waiting times, but also at the later T, the spectral lineshape is quite similar to that seen in experiment (Fig. 3). At later T, (Fig. S15b), however, the amplitude of the 2DES map, thus the strength of the nonlinearity, is much reduced. The dispersive nature of the cross peaks in Fig. S15b arises from the loss of information about the excitation pathways after electronic dephasing. Both incoherent polariton populations and dark states therefore show the same spectral markers. The dark states can be long-lived and may therefore act as efficient traps for the polaritons, competing with their radiative decay. In the absence of such dark states, however, the incoherent polariton populations will rapidly decay radiatively. This rapid radiative decay gives rise to the low amplitude of the 2DES map in Fig. S15b. While the 2DES lineshapes alone do not allow one to unambiguously distinguish between these two kinds of excitations, the long-lived nature and strong amplitude of the 2DES signal for longer waiting times is a distinct signature of the dark state population. Differences between experimentally observed lifetime and those in the 3-COM may thus indicate the presence of additional dark reservoir states, either electronic states of the TMD or of a collective nature, beyond those considered in the simulations.

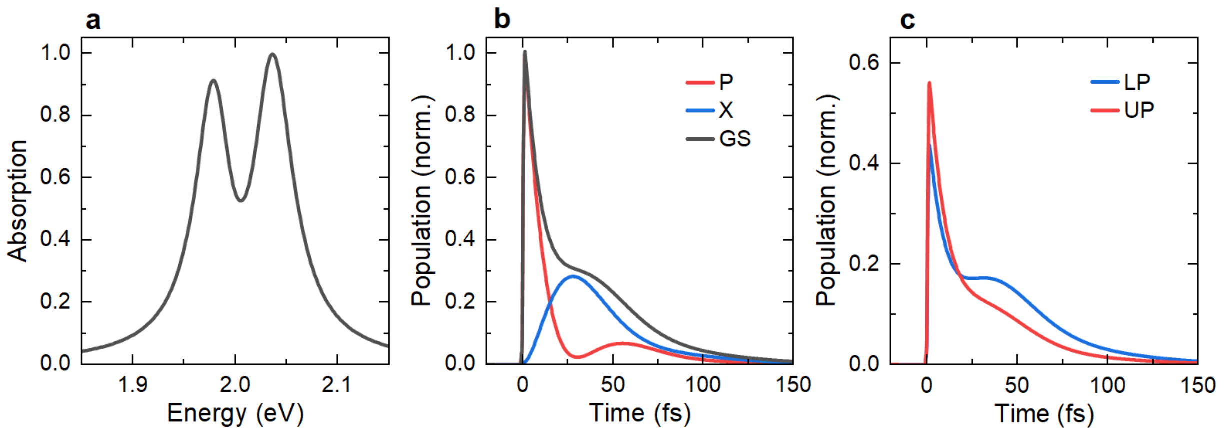

Figure S14: *Simulations using only a single bright exciton coupled to the plasmon.* ***a:*** *linear absorption spectrum* ***b:*** *Population dynamics in the X-P basis.* ***c:*** *Population dynamics in the polariton basis. In both bases, the lack of dark states in the model results in a rapid decay of the excited state population due to radiative damping. After ~100 fs, the population has vanished almost completely, unlike in experiment.*

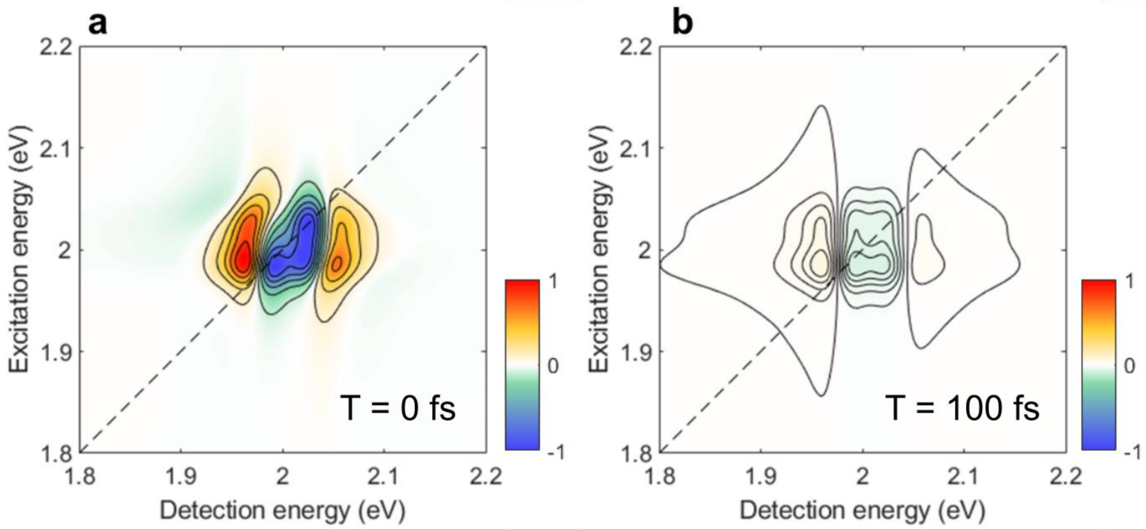

Figure S15: *2DES maps simulated for only considering a single bright exciton for (a) $T = 0$ fs and (b) 100 fs. While the maps show similar line shapes as those displayed in Fig. 4, the 2DES amplitude at $T = 100$ fs vanishes almost completely. The change of the line shape of the 2DES map with waiting time arises due to the rapid decay of coherent polariton excitations.*

Coherent vs. incoherent polaritons

To quantitatively analyze the degree of coherence, thus the transfer from coherent to incoherent polaritons, we compute the purity of the polaritons. For this, we use the excited state density matrix $\hat{\rho}_{ES}(t)$, containing the X and P populations and their coherences. We obtain the degree of coherence via

$$purity = \mathrm{Tr}\left[\left(\frac{\hat{\rho}_{ES}(t)}{\mathrm{Tr}[\hat{\rho}_{ES}(t)]}\right)^2\right]. \tag{S21}$$

The normalization to the total excited state population $\mathrm{Tr}[\hat{\rho}_{ES}(t)]$ ensures that the purity accurately traces the loss of coherence of the off-diagonal density matrix elements. A decay of the excited state population, however, will not affect the normalized purity. For a pure quantum state (fully coherent polaritons), the purity is 1. For a fully incoherent state (no off-diagonal elements in the density matrix between X and P), the purity can reach 0.5 if the diagonal elements have the same amplitude. This would mark fully incoherent polaritons. A loss of purity therefore directly measures the transfer from coherent to incoherent polaritons. Fig. S16 shows the excited-state-normalized purity for the density matrix simulation (blue line) compared to the excited state lifetime (black line). During the short polariton lifetime (exponential decay of ~35 fs), the purity drops from an initial value of 1 (coherent polaritons) after optical excitation to ~0.7 with a decay time of 42 fs, the exciton dephasing time. This shows the rapid loss of coherence and thus explains the changes seen in the 2DES maps in Fig. S15.

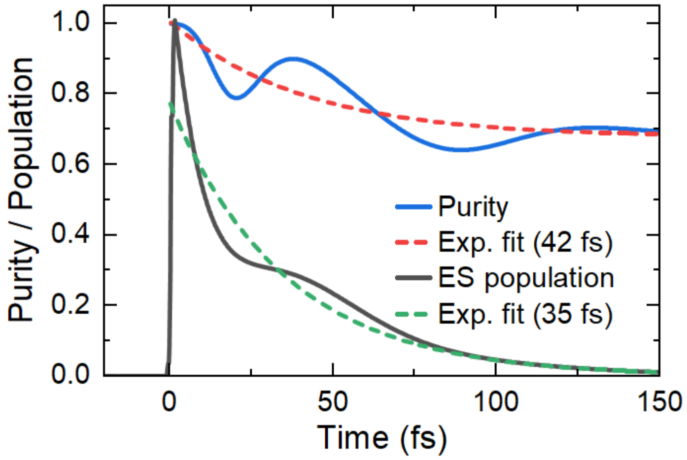

Figure S16: *Analysis of polariton coherence via their purity. After excitation, a purity of 1 marks a quantum mechanically pure (fully coherent) state. Decay of the purity with the dephasing time (40 fs) marks a transfer to an incoherent state, i.e. incoherent polaritons.*

8. Model validation and coupling strength estimation

Estimation of the effective exciton momentum in the vicinity to the nanoslit array

For excitation energies of about 2 eV in the spacer material with $n_{\mathrm{Al_2O_3}} = 1.76$,[11] the photon momentum (wave number) is $k_0 = n_{\mathrm{Al_2O_3}} \frac{\omega}{c} = 0.018/\mathrm{nm}$. The effective exciton momentum is given by

$$q_{\mathrm{eff}} = \frac{\int_{\mathbb{R}} dq_x \, |q_x| |\mathbf{P}_{q_x}^{\mathrm{ex}}|}{\int_{\mathbb{R}} dq_x |\mathbf{P}_{q_x}^{\mathrm{ex}}|} \approx \frac{\int_{\mathbb{R}} dq_x \, |q_x| |\mathbf{E}_{q_x}^{\mathrm{pl}}|}{\int_{\mathbb{R}} dq_x |\mathbf{E}_{q_x}^{\mathrm{pl}}|} \tag{S22}$$

where the momentum distribution of the excitons with wavevector q_x, $\mathbf{P}_{q_x}^{\mathrm{ex}}$, is approximated to follow the Fourier transform of the electric field, $\mathbf{E}_{q_x}^{\mathrm{pl}}$. We obtain $q_{\mathrm{eff}} = 0.03/\mathrm{nm}$ as an expectation value of $|q_x|$ regarding the numeric Fourier transform from the real space FDTD electric field data E_X for the Ag nanoslit array (Fig. S4, left). Therefore, the main contribution of plasmon-coupled excitons lies well outside the light-cone (since $q_{\mathrm{eff}} > k_0$) such that using the 3-COM[16] to distinguish between exciton species inside and outside the lightcone is appropriate.

Coupling strength ratio

We expect that the symmetry of the nanoplasmonic structure coupled to the excitonic layer influences the ratio between coupling to the plasmon of the two effective exciton species. This symmetry of our nanogrid structure, translationally invariant in y-direction, is different from that of ournanoparticle on 1L-TMD structure, considered in our previous derivation of the 3-COM,[16] exhibiting in-plane rotational invariance. We therefore develop a model similar to that in Ref. 16 for infinetly long nanorods coupled to the 1L-TMD. The theory is derived in Sec. 9. Here, we present the result in advance, that is the effective coupling strength ratio from excitons within the lightcone (∘) and excitons outside the lightcone (•) to the plasmon. Applying the parameters from **Table S2** to Eq. (S49) yields

$$\frac{g_{\circ}^{\mathrm{eff}}}{g_{\bullet}^{\mathrm{eff}}} \approx 0.8. \tag{S23}$$

Table S2: *Parameters*

Parameter	Value	Reference
δz	30 nm	
$\varepsilon_{\mathrm{out}} = (n_{\mathrm{Al_2O_3}})^2$	3.1	11
$\hbar\omega_0$	2 eV	

The parameters are chosen in in line with the experimental setup:

• The exciton-plasmon distance $\delta z = (25 + 5)$ nm corresponds to half of the nanogrid depth plus the thickness of the spacer layer.

• The material mediating the electric field responsible for the coupling between nanogrid plasmons and TMD excitons is the spacer layer: $\mathrm{Al_2O_3}$

• The coupling strengths depend on energy but their order of magnitude is not sensitive to changes of about 100 meV such that we choose the average value of about 2 eV.

9. 3-COM for TMD coupled infinitely long metal nanorods

Individual exciton and plasmon harmonic oscillator equations

The harmonic oscillator equations for the 1L-TMD dipole density $\mathbf{P}_{\mathbf{q}_\parallel}^{\mathrm{ex}\circ/\bullet}(\omega)$ in Fourier domain for excitons within the lightcone (∘) and excitons outside the lightcone (•) can be adopted identically from Ref. 16,

$$(\omega^2 + i\gamma_{\circ}^{\mathrm{ex}}\omega - \omega_{\mathrm{ex}\circ}^2)\mathbf{P}_{\mathbf{q}_\parallel}^{\mathrm{ex}\circ}(\omega) = -f^{\mathrm{ex}}(\omega_{\mathrm{ex}\circ})\left(\mathbf{E}_{\mathbf{q}_\parallel}^{\mathrm{pl}}(z_{\mathrm{ex}};\omega) + \mathbf{E}_{\mathbf{q}_\parallel}^{0}(z_{\mathrm{ex}};\omega)\right), \tag{S24}$$

$$\begin{aligned}\left((\omega^2 + i\gamma^{\mathrm{ex}}\omega - \omega_{\mathrm{ex}}^2(q_\parallel))\mathbf{1} - \frac{\omega_{\mathrm{ex}}(q_\parallel)}{\hbar}\frac{|\varphi_0 d|^2}{\varepsilon_0\varepsilon_{\mathrm{out}}} q_\parallel \boldsymbol{\mathcal{U}}_{\mathbf{q}_\parallel}\right)\cdot \mathbf{P}_{\mathbf{q}_\parallel}^{\mathrm{ex}\bullet}(\omega) \\ = -f^{\mathrm{ex}}(\omega_{\mathrm{ex}}(q_\parallel))\left(\mathbf{E}_{\mathbf{q}_\parallel}^{\mathrm{pl}}(z_{\mathrm{ex}};\omega) + \mathbf{E}_{\mathbf{q}_\parallel}^{0}(z_{\mathrm{ex}};\omega)\right),\end{aligned} \tag{S25}$$

with momentum $q_\parallel$ dependent exciton resonance frequencies

$$\hbar\omega_{\mathrm{ex}}(q_\parallel) = \hbar\omega_{\mathrm{ex}\circ} + \frac{\hbar^2 q_\parallel^2}{2M}, \tag{S26}$$

according to the exciton dispersion with exciton mass M, damping constant γ^{ex} arising from phonons and $\gamma_\circ^{\mathrm{ex}}$ that additionally includes radiative damping, dipole element d, exciton wave function φ_0, surrounding permittivity $\varepsilon_{\mathrm{out}}$ and an idempotent matrix

$$\boldsymbol{\mathcal{U}}_{\mathbf{q}_\parallel} = \frac{\mathbf{q}_\parallel \otimes \mathbf{q}_\parallel}{q_\parallel^2}. \tag{S27}$$

The excitation is given by the incident electric field $\mathbf{E}^0_{\mathbf{q}_\parallel}(z_{\mathrm{ex}};\omega)$ and the electric field emitted by the plasmonic structure $\mathbf{E}^{\mathrm{pl}}_{\mathbf{q}_\parallel}(z_{\mathrm{ex}};\omega)$, weighted with the exciton oscillator strength $f^{\mathrm{ex}}(\omega_{\mathrm{ex}}(q_\parallel))$.

On the contrary, the plasmon equation from Ref. 16 must be modified since here $\mathbf{p}^{\mathrm{pl}}$ describes a 1d dipole density of an infinitely long, thin rod,

$$\mathbf{P}^{\mathrm{pl}}(\mathbf{r},\omega) = \mathbf{p}^{\mathrm{pl}}(\omega)\delta(z - z^{\mathrm{pl}})\delta(x - x^{\mathrm{pl}}), \tag{S28}$$

constant in y-direction. For the FDTD simulations, we assume full translational invariance in y-direction for the incident electric field $\mathbf{E}^0$, such that it also applies for $\mathbf{P}^{\mathrm{pl/ex}}(\mathbf{r},\omega)$ and for $\mathbf{E}^{\mathrm{ex}}$ and $\mathbf{E}^{\mathrm{pl}}$. We assume here (without proof) that $\mathbf{p}^{\mathrm{pl}}$ follows the dynamics of a harmonic oscillator:

$$\left(\omega^2 + i\gamma^{\mathrm{pl}}\omega - \omega_{\mathrm{spp}}^2\right)\mathbf{p}^{\mathrm{pl}}(\omega) = -f^{\mathrm{pl}}\left(\mathbf{E}^{\mathrm{ex}\circ}(\mathbf{r}_{\mathrm{pl}};\omega) + \mathbf{E}^{\mathrm{ex}\bullet}(\mathbf{r}_{\mathrm{pl}};\omega) + \mathbf{E}^0(\mathbf{r}_{\mathrm{pl}};\omega)\right) \tag{S29}$$

with some oscillator strength f^{pl}, damping constant γ^{pl} and resonance frequency ω_{spp}.

Emitted electric fields

From Eqs. (S24), (S25) and (S29), excitons and the plasmonic mode are coupled via their emitted electric fields. The y-translational invariance allows us to reduce the dimensionality in the Fourier domain since

$$\mathbf{E}^0_{\mathbf{q}_\parallel} = 2\pi\delta(q_y)\,\mathbf{E}^0_{q_x}, \tag{S30}$$

$$\mathbf{P}^{\mathrm{ex}\circ/\bullet}_{\mathbf{q}_\parallel} = 2\pi\delta(q_y)\,\mathbf{P}^{\mathrm{ex}\circ/\bullet}_{q_x}, \tag{S31}$$

$$\boldsymbol{\mathcal{U}}_{\mathbf{q}_\parallel}|_{q_y=0} = \frac{\mathbf{q}_\parallel \otimes \mathbf{q}_\parallel}{q_\parallel^2} = \begin{pmatrix}1 & 0\\ 0 & 0\end{pmatrix} := \boldsymbol{\mathcal{U}}, \tag{S32}$$

such that the Green's function formalism yields

$$\begin{aligned}\mathbf{E}^{\mathrm{ex}\circ/\bullet}(\mathbf{r}_{\mathrm{pl}};\omega) &= \frac{1}{(2\pi)^2}\int d^2q_\parallel \boldsymbol{G}_{\mathbf{q}_\parallel}(z_{\mathrm{pl}}, z_{\mathrm{ex}};\omega)\cdot \mathbf{P}^{\mathrm{ex}\circ/\bullet}_{\mathbf{q}_\parallel}(\omega)\\ &= \frac{1}{2\pi}\int dq_x \boldsymbol{G}_{q_y=0,q_x}(z_{\mathrm{pl}}, z_{\mathrm{ex}};\omega)\cdot \mathbf{P}^{\mathrm{ex}\circ/\bullet}_{q_x}(\omega),\end{aligned} \tag{S33}$$

$$\mathbf{E}^{\mathrm{pl}}_{\mathbf{q}_\parallel}(z_{\mathrm{ex}};\omega) = 2\pi\delta(q_y)\,\boldsymbol{G}_{q_y=0,q_x}(z_{\mathrm{ex}}, z_{\mathrm{pl}};\omega)\cdot \mathbf{p}^{\mathrm{pl}}(\omega). \tag{S34}$$

Within the lightcone ($q_x < k_0$) we assume $q_x \approx 0$ for the Green's dyadic $\boldsymbol{G}_{\mathbf{q}_\parallel} \rightarrow \boldsymbol{G}_{q_\parallel=0}$, whereas outside the lightcone ($q_x > k_0$) the Green's dyadic is treated as quasi-static, corresponding to $\omega \approx 0$, which we denote by dropping the corresponding dependence. The emitted electric fields simplify to

$$\mathbf{E}^{\mathrm{ex}\circ}(\mathbf{r}_{\mathrm{pl}};\omega) = \frac{1}{2\pi}\boldsymbol{G}_{q_\parallel=0}(z_{\mathrm{pl}}, z_{\mathrm{ex}};\omega)\cdot\int_{|q_x|<k_0} dq_x \mathbf{P}_{q_x}^{\mathrm{ex}\circ}(\omega), \tag{S35}$$

$$\mathbf{E}^{\mathrm{ex}\bullet}(\mathbf{r}_{\mathrm{pl}};\omega) = \frac{1}{2\pi}\int dq_x\, \boldsymbol{G}_{q_y=0,q_x}(z_{\mathrm{pl}}, z_{\mathrm{ex}})\cdot \mathbf{P}_{q_x}^{\mathrm{ex}\bullet}(\omega), \tag{S36}$$

$$\mathbf{E}_{\mathbf{q}_\parallel}^{\mathrm{pl}}(z_{\mathrm{ex}};\omega) = 2\pi\delta(q_y)\, \boldsymbol{G}_{q_y=0,q_x}(z_{\mathrm{ex}}, z_{\mathrm{pl}};\omega)\cdot \mathbf{p}^{\mathrm{pl}}(\omega). \tag{S37}$$

Since we assume a non-evanescent incident electric field from the far-field, we furthermore know that it vanishes outside the lightcone $\mathbf{E}_{q_\parallel > k_0}^{0} = 0$.

Coupled equations

Inserting the emitted electric fields, Eqs. (S35), (S36) and (S37), into the harmonic oscillator Eqs. (S24), (S25) and (S29), yields

$$\begin{aligned}(\omega^2 + i\gamma_\circ^{\mathrm{ex}}\omega - \omega_{\mathrm{ex}\circ}^2)\mathbf{P}_{q_x}^{\mathrm{ex}\circ}(\omega) \\ = -f^{\mathrm{ex}}(\omega_{\mathrm{ex}\circ})\big(\boldsymbol{G}_{q_\parallel=0}(z_{\mathrm{ex}}, z_{\mathrm{pl}};\omega)\cdot \mathbf{p}^{\mathrm{pl}}(\omega) + \mathbf{E}_{q_x}^{0}(z_{\mathrm{ex}};\omega)\big),\end{aligned} \tag{S38}$$

$$\begin{aligned}\left((\omega^2 + i\gamma^{\mathrm{ex}}\omega - \omega_{\mathrm{ex}}^2(q_x))\mathbf{1} - \frac{\omega_{\mathrm{ex}}(q_x)}{\hbar}\frac{|\varphi_0 d|^2}{\varepsilon_0\varepsilon_{\mathrm{out}}} q_x \boldsymbol{\mathcal{U}}\right)\cdot \mathbf{P}_{q_x}^{\mathrm{ex}\bullet}(\omega) \\ = -f^{\mathrm{ex}}(\omega_{\mathrm{ex}}(q_x))\, \boldsymbol{G}_{q_y=0,q_x}(z_{\mathrm{ex}}, z_{\mathrm{pl}})\cdot \mathbf{p}^{\mathrm{pl}}(\omega),\end{aligned} \tag{S39}$$

and

$$\begin{aligned}\big(\omega^2 + i\gamma^{\mathrm{pl}}\omega - \omega_{\mathrm{spp}}^2\big)\mathbf{p}^{\mathrm{pl}}(\omega) \\ = -f^{\mathrm{pl}}\Bigg(\frac{1}{2\pi}\boldsymbol{G}_{q_\parallel=0}\big(z_{\mathrm{pl}}, z_{\mathrm{ex}};\omega\big)\cdot\int_{|q_x|<k_0} dq_x \mathbf{P}_{q_x}^{\mathrm{ex}\circ}(\omega) \\ + \frac{1}{2\pi}\int dq_x\, \boldsymbol{G}_{q_y=0,q_x}(z_{\mathrm{pl}}, z_{\mathrm{ex}})\cdot \mathbf{P}_{q_x}^{\mathrm{ex}\bullet}(\omega) + \mathbf{E}^0(\mathbf{r}_{\mathrm{pl}};\omega)\Bigg)\end{aligned} \tag{S40}$$

Now we apply a number of approximations and a similar procedure as in Ref. 16:

1. Identify a single representative exciton mode within the lightcone as an average:

$$\mathbf{P}^{\mathrm{ex}\circ} = \frac{1}{2k_0}\int_{|q_x|<k_0} dq_x \mathbf{P}_{q_x}^{\mathrm{ex}\circ}. \tag{S41}$$

2. Insert the $q_\parallel = 0$ Green's dyadic inside the light cone for the homogeneous dielectric environment

$$\boldsymbol{G}_{q_\parallel=0}(z^{\mathrm{ex}}, z^{\mathrm{pl}};\omega) = \frac{i}{2\varepsilon_0\varepsilon_{\mathrm{out}}}\, k_0 e^{ik_0\delta z}, \tag{S42}$$

with $\delta z = |z^{\text{ex}} - z^{\text{pl}}|$ and the quasi-static Green's dyadic

$$\boldsymbol{G}_{q_y=0,q_x}(z^{\text{ex}}, z^{\text{pl}}) = \frac{-1}{2\varepsilon_0\varepsilon_{\text{out}}} \, |q_x| e^{-|q_x|\delta z} \boldsymbol{\mathcal{U}}. \tag{S43}$$

3. Evaluate the exciton dispersion outside the lightcone at effective momentum $\omega_{\text{ex}}(q_x) \approx \omega_{\text{ex}}(q_{\text{eff}}) = \text{const}$ and the electric field-mediated exciton self-interaction at $q_x = q_{\text{eff}}$. The evaluation of Eq. (S26) with the exciton mass $M = 3.5$ eV fs^{2}nm^{-2} for WS_2 [41] and $q_{\text{eff}} = 0.03$ nm, see Sec. 8, yields $\omega_{\text{ex}}(q_{\text{eff}}) \approx \omega_{\text{ex}\circ}$ with deviations of below 0.1 meV. The electric field-mediated exciton self-interaction outside the lightcone (prefactor of $\boldsymbol{\mathcal{U}}$ in Eq. (S39)) is similarly negligible (deviations of about 1 meV).
4. Apply a separation ansatz for excitons outside the lightcone:

$$\mathbf{P}_{q_x}^{\text{ex}\bullet}(\omega) = \delta z \, |q_x| e^{-|q_x|\delta z} \boldsymbol{\mathcal{U}} \cdot \mathbf{P}^{\text{ex}\bullet}(\omega). \tag{S44}$$

5. In the plasmon equation, the following integral arises from the coupling to excitons outside the lightcone:

$$\int_{-\infty}^{\infty} dq_x \, \delta z \, |q_x|^2 e^{-2|q_x|\delta z} = \frac{1}{2} \, \delta z^{-2}. \tag{S45}$$

Final set of 3 coupled oscillator equations

Excitons within the lightcone:

$$\begin{aligned}(\omega^2 + i\gamma_\circ^{\text{ex}}\omega - \omega_{\text{ex}\circ}^2)\mathbf{P}^{\text{ex}\circ}(\omega) + \underbrace{f^{\text{ex}} \frac{i}{2\varepsilon_0\varepsilon_{\text{out}}} \, k_0 e^{ik_0\delta z}}_{g_\circ^{\text{ex}}} \, \mathbf{p}^{\text{pl}}(\omega) \\ = -f^{\text{ex}} \frac{1}{2k_0} \int_{|q_x|<k_0} dq_x \, \mathbf{E}_{q_x}^0(z_{\text{ex}}; \omega),\end{aligned} \tag{S46}$$

Excitons outside the lightcone:

$$(\omega^2 + i\gamma^{\text{ex}}\omega - \omega_{\text{ex}\circ}^2)\mathbf{P}^{\text{ex}\bullet}(\omega) - \underbrace{\frac{f^{\text{ex}}}{2\varepsilon_0\varepsilon_{\text{out}}\delta z}}_{g_\bullet^{\text{ex}}} \, \mathbf{p}^{\text{pl}}(\omega) = 0, \tag{S47}$$

Surface plasmon polariton:

$$\begin{aligned}\left(\omega^2 + i\gamma^{\text{pl}}\omega - \omega_{\text{spp}}^2\right)\mathbf{p}^{\text{pl}}(\omega) + \underbrace{f^{\text{pl}} \frac{2i}{4\pi\varepsilon_0\varepsilon_{\text{out}}} \, k_0^2 e^{ik_0\delta z}}_{g_\circ^{\text{pl}}} \, \mathbf{P}^{\text{ex}\circ}(\omega) \\ \underbrace{-f^{\text{pl}} \frac{1}{2\delta z^2} \frac{1}{4\pi\varepsilon_0\varepsilon_{\text{out}}}}_{g_\bullet^{\text{pl}}} \, \boldsymbol{\mathcal{U}} \cdot \mathbf{P}^{\text{ex}\bullet}(\omega) = -f^{\text{pl}}\mathbf{E}^0(\mathbf{r}_{\text{pl}}; \omega).\end{aligned} \tag{S48}$$

This set of Eqs. (S46), (S47) and (S48) constitutes the 3-COM modified for the 1L-TMD coupled to a plasmonic system featuring in-plane translational invariance in one direction (y). Different from Ref. 16, here we remain in the frequency domain, since in the time domain the coupling would involve time derivatives of exciton and plasmon dipole densities that, however, are sufficiently approximated by the carrier frequency of the corresponding elementary excitation: $\partial_t \leftrightarrow -i\omega \approx -i\,\omega_{ex\circ}, -i\,\omega_{spp}$. The time delays resulting in time domain from the complex exponential prefactors can be disregarded for distances as small as $\delta z = 30$ nm $\ll \lambda$.

We identify the prefactors as the coupling strengths $g_\circ^{\mathrm{ex}}, g_\bullet^{\mathrm{ex}}, g_\circ^{\mathrm{pl}}$, and $g_\bullet^{\mathrm{pl}}$. Similar coupling dynamics as in Eqs. (S46), (S47) and (S48) would be obtained without distinguishing between $g_\circ^{\mathrm{ex}}$ and $g_\circ^{\mathrm{pl}}$ (and similarly $g_\bullet^{\mathrm{ex}}$ and $g_\bullet^{\mathrm{pl}}$) by instead using effective coupling constants $g_\circ^{\mathrm{eff}} = g_\circ^{\mathrm{ex}} g_\circ^{\mathrm{pl}}$ and $g_\bullet^{\mathrm{eff}} = g_\bullet^{\mathrm{ex}} g_\bullet^{\mathrm{pl}}$.

We find for their ratio

$$\left(\frac{g_\circ^{\mathrm{eff}}}{g_\bullet^{\mathrm{eff}}}\right)_{\mathrm{rod-TMD}} = 2\sqrt{k_0^3 \delta z^3} = \sqrt{\frac{3}{2k_0\delta z}} \left(\frac{g_\circ^{\mathrm{eff}}}{g_\bullet^{\mathrm{eff}}}\right)_{\mathrm{nanoparticle-TMD}}. \tag{S49}$$

Due to the lack of field localization in y-direction, this coupling strength ratio is higher than for nanoparticle-TMD coupling.

10. Deviation from bosonic excitons - Pauli-blocking

In the coupled oscillator model, it was assumed, that excitonic creation and annihilation operators b_X, $b_X^\dagger$ obey the standard bosonic commutation relation $[b_X, b_{X'}^\dagger]_- = \delta_{X,X'}$. However, excitons are not perfect bosons and obey the commutation relation:

$$\begin{aligned}[b_X, b_{X'}^\dagger]_- = \delta_{X,X'} &- \sum_{k_e \tilde{k}_e k_h} \Psi_X(k_e, k_h)\Psi_{X'}^*(\tilde{k}_e, k_h) e_{k_e}^\dagger e_{\tilde{k}_e} \\ &- \sum_{k_h \tilde{k}_h k_e} \Psi_X(k_e, k_h)\Psi_{X'}^*(k_e, \tilde{k}_h) h_{k_h}^\dagger h_{\tilde{k}_h},\end{aligned} \tag{S50}$$

where Ψ_X are exciton wave functions in momentum spaces for the electron and the hole forming the exciton and $e^\dagger$, e and $h^\dagger$, h the electron and hole creation and annihilation operators. If we assume that the deviation from the commutation relation mainly comes from free carriers created during the excitation in addition to the excitons (introducing a fifth-order nonlinear susceptibility χ_5), we approximate the electron creation and annihilation operator with an expectation value, assuming furthermore a spatially homogeneous distribution:

$$\begin{aligned}[b_X, b_{X'}^\dagger]_- \approx \delta_{X,X'} &- \sum_{k_e k_h} \Psi_X(k_e, k_h)\Psi_{X'}^*(\tilde{k}_e, k_h) \langle e_{k_e}^\dagger e_{\tilde{k}_e} \rangle \\ &- \sum_{k_h k_e} \Psi_X(k_e, k_h)\Psi_{X'}^*(k_e, k_h) \langle h_{k_h}^\dagger h_{k_h} \rangle,\end{aligned} \tag{S51}$$

To first approximation, the electron and hole densities scale quadratically in the exciting field strength E. This introduces a time dependence in the commutation relations with a reduction of the commutator value which is proportional to $|E|^2$:

$$[b_X, b_{X'}^\dagger]_{\approx}\delta_{X,X'}(1 - c|E|^2) \quad \text{(S52)}$$

Here, $|E|$ and c are effective time-dependent quantities. This shows that the commutation relation value is reduced from 1 after the initial excitation, which may suggest that excitation of an electron-hole complex of higher order may have a reduced electron-light coupling element compared to the earlier interaction, where no additional electron-hole density is built up.

11. Additional data

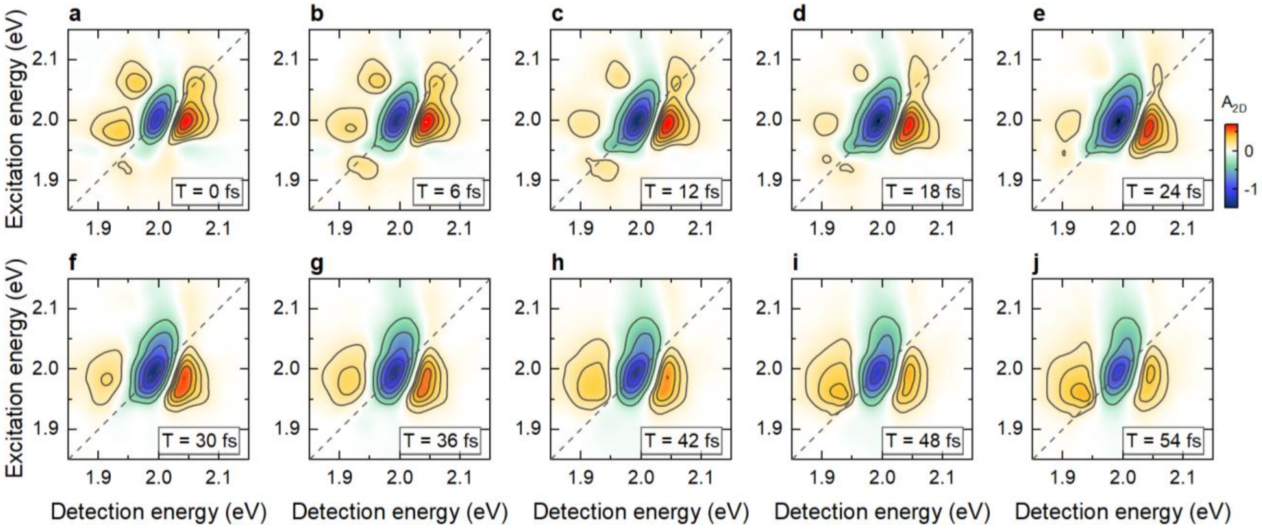

Figure S17: *Additional 2DES maps to those shown in Fig. 3. For early waiting times up to ~24 fs, sub-peak structure due to coherent polaritons can be discerned. For later waiting times, the 2DES maps develop into vertical stripes, a signature for a transfer from coherent polaritons to incoherent polaritons and dark states.*

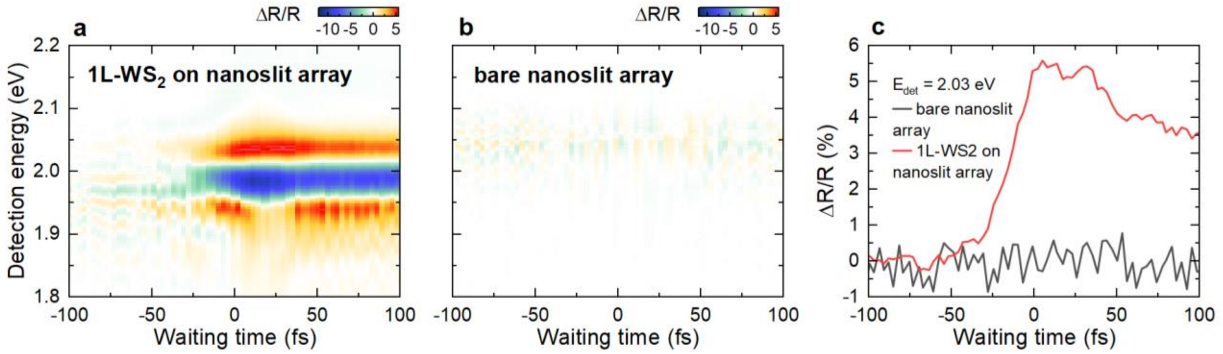

Figure S18: *Study of nanoslit array nonlinearity, recorded with 10 µJ/cm² fluence and 5° incidence angle. Data consists of only a single pump-probe scan and is contaminated by interference with scattered pump light manifesting itself as spectral oscillations.* ***a:*** *Map for 1L-WS_2 on nanoslit array.* ***b:*** *Map recorded for a nanoslit array without 1L-WS_2 under identical experimental conditions. Only weak signal due to pump*

scattering can be seen, mostly around 2.05 eV, the resonance position of the SPP without 1L-WS_2 (see Fig. S3). ***c:*** *Crosscuts at 2.03 eV confirm that no significant SPP nonlinearity is recorded beyond the linear scattering contribution.*

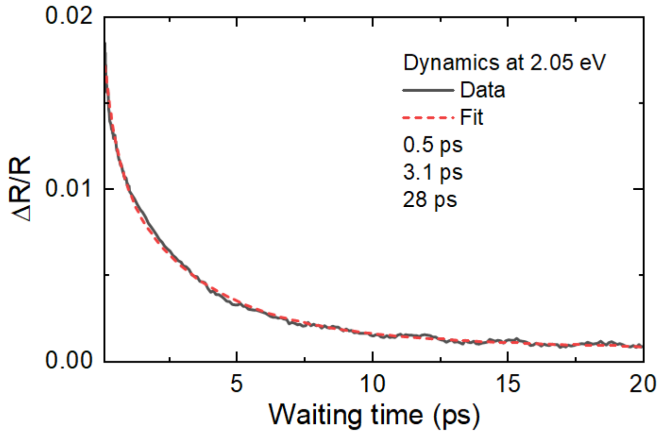

Figure S19: *Dynamics for the hybrid structure measured up to 20 ps, taken at 2.05 eV (black line) with 3-exponential fit (red line). The time constants are 0.5 ps, 3.1 ps and 28 ps. Faint oscillations seen on the data are an artifact due to a periodic error of the retro reflector stage.*

12. References

(1) Brongersma, M. L.; Halas, N. J.; Nordlander, P. Plasmon-induced hot carrier science and technology. *Nature Nanotechnology* **2015**, *10* (1), 25-34.

(2) Li, Z.; Xiao, Y.; Gong, Y.; Wang, Z.; Kang, Y.; Zu, S.; Ajayan, P. M.; Nordlander, P.; Fang, Z. Active Light Control of the MoS2 Monolayer Exciton Binding Energy. *ACS Nano* **2015**, *9* (10), 10158-10164.

(3) Xu, C.; Yong, H. W.; He, J.; Long, R.; Cadore, A. R.; Paradisanos, I.; Ott, A. K.; Soavi, G.; Tongay, S.; Cerullo, G. Weak distance dependence of hot-electron-transfer rates at the interface between monolayer MoS2 and gold. *ACS nano* **2020**, *15* (1), 819-828.

(4) Novoselov, K. S.; Jiang, D.; Schedin, F.; Booth, T.; Khotkevich, V.; Morozov, S.; Geim, A. K. Two-dimensional atomic crystals. *Proceedings of the National Academy of Sciences* **2005**, *102* (30), 10451-10453.

(5) Casiraghi, C.; Hartschuh, A.; Lidorikis, E.; Qian, H.; Harutyunyan, H.; Gokus, T.; Novoselov, K. S.; Ferrari, A. Rayleigh imaging of graphene and graphene layers. *Nano letters* **2007**, *7* (9), 2711-2717.

(6) Cadore, A. R.; Rosa, B. L. T.; Paradisanos; Mignuzzi, S.; De Fazio, D.; Alexeev, E. M.; Dagkli, A.; Muench, J. E.; Kakavelakis, G.; Shinde, S. M.; Yoon, D.; Tongay, S.; Watanabe, K.; Taniguchi, T.; Lidorikis, E.; Goykhman; Soavi, G.; Ferrari, A. C. Monolayer WS2 electro- and photo-luminescence enhancement by TFSI treatment. *2d Materials* **2024**, *11* (2), 025017.

(7) Ropers, C.; Park, D. J.; Stibenz, G.; Steinmeyer, G.; Kim, J.; Kim, D. S.; Lienau, C. Femtosecond light transmission and subradiant damping in plasmonic crystals. *Physical Review Letters* **2005**, *94* (11), 113901.

(8) Kim, D. S.; Hohng, S. C.; Malyarchuk, V.; Yoon, Y. C.; Ahn, Y. H.; Yee, K. J.; Park, J. W.; Kim, J.; Park, Q. H.; Lienau, C. Microscopic origin of surface-plasmon radiation in plasmonic band-gap nanostructures. *Physical Review Letters* **2003**, *91* (14), 143901.

(9) Liang, B.; Bai, M.; Ma, H.; Ou, N. M.; Miao, J. G. Wideband Analysis of Periodic Structures at Oblique Incidence by Material Independent FDTD Algorithm. *Ieee Transactions on Antennas and Propagation* **2014**, *62* (1), 354-360.

(10) Yang, H. H. U.; D'Archangel, J.; Sundheimer, M. L.; Tucker, E.; Boreman, G. D.; Raschke, M. B. Optical dielectric function of silver. *Physical Review B* **2015**, *91* (23), 235137.

(11) Malitson, I. H.; Dodge, M. J. Refractive-Index and Birefringence of Synthetic Sapphire. *Journal of the Optical Society of America* **1972**, *62* (11), 1405-1405.

(12) Timmer, D.; Gittinger, M.; Quenzel, T.; Stephan, S.; Zhang, Y.; Schumacher, M. F.; Lützen, A.; Silies, M.; Tretiak, S.; Zhong, J.-H.; De Sio, A.; Lienau, C. Plasmon mediated coherent population oscillations in molecular aggregates. *Nature Communications* **2023**, *14* (1), 8035.

(13) Li, Y.; Chernikov, A.; Zhang, X.; Rigosi, A.; Hill, H. M.; van der Zande, A. M.; Chenet, D. A.; Shih, E.-M.; Hone, J.; Heinz, T. F. Measurement of the optical dielectric function of monolayer transition-metal dichalcogenides: MoS2, MoSe2, WS2, and WSe2. *Physical Review B* **2014**, *90* (20), 205422.

(14) Timmer, D.; Gittinger, M.; Quenzel, T.; Cadore, A. R.; Rosa, B. L. T.; Li, W. S.; Soavi, G.; Lünemann, D. C.; Stephan, S.; Silies, M.; Schulz, T.; Steinhoff, A.; Jahnke, F.; Cerullo, G.; Ferrari, A. C.; De Sio, A.; Lienau, C. Ultrafast Coherent Exciton Couplings and Many-Body Interactions in Monolayer WS2. *Nano Letters* **2024**, *24* (26), 8117-8125.

(15) Greten, L.; Salzwedel, R.; Göde, T.; Greten, D.; Reich, S.; Hughes, S.; Selig, M.; Knorr, A. Strong Coupling of Two-Dimensional Excitons and Plasmonic Photonic Crystals: Microscopic Theory Reveals Triplet Spectra. *Acs Photonics* **2024**, *11* (4), 1396-1411.

(16) Greten, L.; Salzwedel, R.; Schutsch, D.; Knorr, A. Microscopic theory for a minimal oscillator model of exciton-plasmon coupling in hybrids of two-dimensional semiconductors and metal nanoparticles. *Physical Review B* **2025**, *111* (20), 205438.

(17) Törmä, P.; Barnes, W. L. Strong coupling between surface plasmon polaritons and emitters: a review. *Reports on Progress in Physics* **2014**, *78* (1), 013901.

(18) Vasa, P.; Lienau, C. Strong Light–Matter Interaction in Quantum Emitter/Metal Hybrid Nanostructures. *ACS Photonics* **2018**, *5* (1), 2-23.

(19) Garraway, B. M. The Dicke model in quantum optics: Dicke model revisited. *Philosophical Transactions of the Royal Society A: Mathematical, Physical and Engineering Sciences* **2011**, *369* (1939), 1137-1155.

(20) Gallego-Valencia, D.; Mewes, L.; Feist, J.; Sanz-Vicario, J. L. Coherent multidimensional spectroscopy in polariton systems. *Physical Review A* **2024**, *109* (6), 063704.

(21) DelPo, C. A.; Kudisch, B.; Park, K. H.; Khan, S. U. Z.; Fassioli, F.; Fausti, D.; Rand, B. P.; Scholes, G. D. Polariton Transitions in Femtosecond Transient Absorption Studies of Ultrastrong Light-Molecule Coupling. *Journal of Physical Chemistry Letters* **2020**, *11* (7), 2667-2674.

(22) Grupp, A.; Budweg, A.; Fischer, M. P.; Allerbeck, J.; Soavi, G.; Leitenstorfer, A.; Brida, D. Broadly tunable ultrafast pump-probe system operating at multi-kHz repetition rate. *Journal of Optics* **2018**, *20* (1), 014005.

(23) Brida, D.; Manzoni, C.; Cerullo, G. Phase-locked pulses for two-dimensional spectroscopy by a birefringent delay line. *Optics Letters* **2012**, *37* (15), 3027-3029.

(24) Timmer, D.; Lünemann, D. C.; Riese, S.; De Sio, A.; Lienau, C. Full visible range two-dimensional electronic spectroscopy with high time resolution. *Optics Express* **2024**, *32* (1), 835-847.

(25) Réhault, J.; Maiuri, M.; Oriana, A.; Cerullo, G. Two-dimensional electronic spectroscopy with birefringent wedges. *Review of Scientific Instruments* **2014**, *85* (12), 123107.

(26) Palmieri, B.; Abramavicius, D.; Mukamel, S. Lindblad equations for strongly coupled populations and coherences in photosynthetic complexes. *Journal of Chemical Physics* **2009**, *130* (20), 204512.

(27) Breuer, H.-P.; Petruccione, F. *The theory of open quantum systems*; Oxford University Press, 2002.

(28) Wang, G.; Chernikov, A.; Glazov, M. M.; Heinz, T. F.; Marie, X.; Amand, T.; Urbaszek, B. Colloquium: Excitons in atomically thin transition metal dichalcogenides. *Reviews of Modern Physics* **2018**, *90* (2), 021001.

(29) Vasa, P.; Wang, W.; Pomraenke, R.; Lammers, M.; Maiuri, M.; Manzoni, C.; Cerullo, G.; Lienau, C. Real-time observation of ultrafast Rabi oscillations between excitons and plasmons in metal nanostructures with J-aggregates. *Nature Photonics* **2013**, *7* (2), 128-132.

(30) Moody, G.; Dass, C. K.; Hao, K.; Chen, C. H.; Li, L. J.; Singh, A.; Tran, K.; Clark, G.; Xu, X. D.; Berghäuser, G.; Malic, E.; Knorr, A.; Li, X. Q. Intrinsic homogeneous linewidth and broadening mechanisms of excitons in monolayer transition metal dichalcogenides. *Nature Communications* **2015**, *6*, 8315.

(31) Zhong, J. H.; Vogelsang, J.; Yi, J. M.; Wang, D.; Wittenbecher, L.; Mikaelsson, S.; Korte, A.; Chimeh, A.; Arnold, C. L.; Schaaf, P.; Runge, E.; Huillier, A. L.; Mikkelsen, A.; Lienau, C. Nonlinear plasmon-exciton coupling enhances sum-frequency generation from a hybrid metal/semiconductor nanostructure. *Nature Communications* **2020**, *11* (1), 1464.

(32) Trovatello, C.; Katsch, F.; Li, Q.; Zhu, X.; Knorr, A.; Cerullo, G.; Dal Conte, S. Disentangling Many-Body Effects in the Coherent Optical Response of 2D Semiconductors. *Nano Letters* **2022**, *22* (13), 5322-5329.

(33) Katsch, F.; Selig, M.; Knorr, A. Exciton-Scattering-Induced Dephasing in Two-Dimensional Semiconductors. *Physical Review Letters* **2020**, *124* (25), 257402.

(34) Tang, Y. X.; Zhang, Y. B.; Liu, Q. R.; Wei, K.; Cheng, X. A.; Shi, L.; Jiang, T. Interacting plexcitons for designed ultrafast optical nonlinearity in a monolayer semiconductor. *Light-Science & Applications* **2022**, *11* (1), 94.

(35) Katsch, F.; Selig, M.; Knorr, A. Theory of coherent pump–probe spectroscopy in monolayer transition metal dichalcogenides. *2D Materials* **2019**, *7* (1), 015021.

(36) Policht, V. R.; Proscia, N. V.; Cunningham, P. D. Insight into exciton polaritons of two-dimensional transition metal dichalcogenides with time-resolved spectroscopy. *MRS Communications* **2025**, 1-20.

(37) Genco, A.; Louca, C.; Cruciano, C.; Song, K. W.; Trovatello, C.; Di Blasio, G.; Sansone, G.; Randerson, S. A.; Claronino, P.; Georgiou, K.; Jayaprakash, R.; Watanabe, K.; Taniguchi, T.; Lidzey, D. G.; Kyriienko, O.; Dal Conte, S.; Tartakovskii, A. I.; Cerullo, G. Femtosecond switching of strong light-matter interactions in microcavities with two-dimensional semiconductors. *Nature Communications* **2025**, *16* (1), 6490.

(38) Emmanuele, R. P. A.; Sich, M.; Kyriienko, O.; Shahnazaryan, V.; Withers, F.; Catanzaro, A.; Walker, P. M.; Benimetskiy, F. A.; Skolnick, M. S.; Tartakovskii, A. I.; Shelykh, I. A.; Krizhanovskii, D. N. Highly nonlinear trion-polaritons in a monolayer semiconductor. *Nature Communications* **2020**, *11* (1), 3589.

(39) Li, H.; Lomsadze, B.; Moody, G.; Smallwood, C.; Cundiff, S. *Optical multidimensional coherent spectroscopy*; Oxford University Press, 2023.

(40) Tavis, M.; Cummings, F. W. Exact solution for an N-molecule—radiation-field Hamiltonian. *Physical Review* **1968**, *170* (2), 379.

(41) Kormányos, A.; Burkard, G.; Gmitra, M.; Fabian, J.; Zólyomi, V.; Drummond, N. D.; Fal'ko, V. k·p theory for two-dimensional transition metal dichalcogenide semiconductors. *2D Materials* **2015**, *2* (2), 022001.
